# Gene doctoring: a method for recombineering in laboratory and pathogenic *Escherichia coli *strains

**DOI:** 10.1186/1471-2180-9-252

**Published:** 2009-12-09

**Authors:** David J Lee, Lewis EH Bingle, Karin Heurlier, Mark J Pallen, Charles W Penn, Stephen JW Busby, Jon L Hobman

**Affiliations:** 1 School of Biosciences, University of Birmingham, Birmingham B15 2TT, UK; 2 Division of Immunity and Infection, School of Medicine, University of Birmingham, Birmingham B15 2TT, UK; 3 School of Biosciences, University of Nottingham, Sutton Bonington campus, Sutton Bonington, Loughborough, LE12 5RD, UK

## Abstract

**Background:**

Homologous recombination mediated by the λ-Red genes is a common method for making chromosomal modifications in *Escherichia coli*. Several protocols have been developed that differ in the mechanisms by which DNA, carrying regions homologous to the chromosome, are delivered into the cell. A common technique is to electroporate linear DNA fragments into cells. Alternatively, DNA fragments are generated *in vivo *by digestion of a donor plasmid with a nuclease that does not cleave the host genome. In both cases the λ-Red gene products recombine homologous regions carried on the linear DNA fragments with the chromosome. We have successfully used both techniques to generate chromosomal mutations in *E. coli *K-12 strains. However, we have had limited success with these λ-Red based recombination techniques in pathogenic *E. coli *strains, which has led us to develop an enhanced protocol for recombineering in such strains.

**Results:**

Our goal was to develop a high-throughput recombineering system, primarily for the coupling of genes to epitope tags, which could also be used for deletion of genes in both pathogenic and K-12 *E. coli *strains. To that end we have designed a series of donor plasmids for use with the λ-Red recombination system, which when cleaved *in vivo *by the I-SceI meganuclease generate a discrete linear DNA fragment, allowing for C-terminal tagging of chromosomal genes with a 6 × His, 3 × FLAG, 4 × ProteinA or GFP tag or for the deletion of chromosomal regions. We have enhanced existing protocols and technologies by inclusion of a cassette conferring kanamycin resistance and, crucially, by including the *sacB *gene on the donor plasmid, so that all but true recombinants are counter-selected on kanamycin and sucrose containing media, thus eliminating the need for extensive screening. This method has the added advantage of limiting the exposure of cells to the potential damaging effects of the λ-Red system, which can lead to unwanted secondary alterations to the chromosome.

**Conclusion:**

We have developed a counter-selective recombineering technique for epitope tagging or for deleting genes in *E. coli*. We have demonstrated the versatility of the technique by modifying the chromosome of the enterohaemorrhagic O157:H7 (EHEC), uropathogenic CFT073 (UPEC), enteroaggregative O42 (EAEC) and enterotoxigenic H10407 (ETEC) *E. coli *strains as well as in K-12 laboratory strains.

## Background

The λ-Red recombinase system can be used to introduce mutations, deletions, or insertions into the *E. coli *chromosome by recombining regions of homology carried on short single-stranded oligonucleotides or large double-stranded DNA molecules [1]. The λ-Red system consists of three proteins, the *gam*, *exo *and *bet *gene products. When expressed in the cell the Gam protein protects linear double stranded DNA from degradation by the host RecBCD complex. The Exo protein generates single stranded DNA overhangs, which are substrates for recombination, catalyzed by the Bet protein, with homologous regions of the chromosome [2-7].

Several λ-Red recombineering techniques have been developed: Two in particular are of note, which differ in the way that the target DNA is delivered into the cell. The first technique, and arguably the most widely used, was first described by Murphy [5] and later refined by Datsenko and Wanner [2]. In this method a plasmid is used to express the λ-Red genes from an arabinose inducible promoter. Strains expressing λ-Red are transformed, by electroporation, with a dsDNA PCR product carrying an antibiotic cassette flanked by short regions of homology to the target gene. λ-Red mediated recombination occurs, resulting in replacement of the targeted gene by an antibiotic resistance cassette. This cassette can then be excised by FLP recombinase leaving a ~ 80 bp DNA scar in place of the target gene.

The second technique, "gene gorging", designed by Herring and co-workers [4], is a two plasmid method that also utilizes the λ-Red system to generate recombinants. Gene gorging eliminates the need to electroporate a dsDNA fragment into cells, by supplying the regions of homology to the target gene on a donor plasmid that also contains a DNA recognition site for the *Saccharomyces cerevisiae *I-SceI endonuclease. The donor plasmid and the recombineering plasmid, pACBSR (which carries the λ-Red and I-SceI endonuclease genes, under the control of an *araBAD *promoter), are transformed into the recipient strain. Upon arabinose induction, I-SceI cleaves the donor plasmid, providing a linear dsDNA target for the λ-Red system. The obvious advantage of this system is that multiple copies of the homologous DNA are present in the bacterial cell, which increases the number of potential recombination events. The frequency of recombination for gene gorging is reported to be 1-15%, eliminating the absolute requirement for an antibiotic resistance marker to select for recombinants.

We have used both systems for making gene knockouts and gene fusions in laboratory *E. coli *strains. However, we have had less success with these methods in pathogenic strains such as the O157:H7 Sakai strain [8], and virtually no success in the CFT073 UPEC [9], the O42 EAEC [10] and the H10407 ETEC [11] strains. Since techniques such as transduction by P1 phage are incompatible with many pathogenic strains, due to extensive surface antigens that block access to the phage receptor [12], gene deletions have to be made directly in the strain.

Our aim in this study was to establish a high-throughput recombineering system, with particular emphasis on the ability to couple epitope tags to genes, which is compatible, without modification, for use in a wide range of laboratory and wild-type *E. coli *strains. We have achieved this by enhancing the two-plasmid system of Herring and co-workers, making three key modifications. First, a set of donor plasmids have been generated that readily facilitate the deletion of genes or the C-terminal coupling of genes to epitope tags. Second, the inclusion of the *sacB *gene on the donor plasmid allows for the counter-selection of all but true recombinants. Third, the inclusion of an I-SceI recognition site on a derivative of the recombineering plasmid, called pACBSCE means that the plasmid is effectively 'self-cleaving' upon induction of I-SceI and λ Red genes. Hence cells receive a burst of λ-Red before pACBSCE is lost, which is sufficient to induce recombination but limits the exposure of cells to λ-Red function. Prolonged exposure to λ-Red activity is undesirable as it can cause unwanted secondary chromosomal modifications in both K-12 and pathogenic *E. coli *strains [13-15]. We have termed this method Gene Doctoring, abbreviated to G-DOC (*G*ene *D*eletion *O*r *C*oupling), and we have demonstrated its versatility by deleting and coupling genes to epitope tags in pathogenic and laboratory *E. coli *strains.

## Results and Discussion

### Current techniques for recombineering in laboratory and pathogenic *Escherichia coli *strains

#### A. electroporation of linear DNA fragments

The method first described by Murphy [5], later refined by Datsenko and Wanner [2], of electroporating linear double stranded DNA fragments into cells that are then targets for homologous recombination by the λ-Red system, is reported to promote a very low recombination efficiency in *E. coli *K-12 strains: approximately 1 in every 3.5 × 10^6 ^*E. coli *K-12 MG1655 cells that survive electroporation [4]. Despite this low frequency, we routinely identify between 10-50 MG1655 recombinants per experiment, however, since we use approximately 1 × 10^9 ^MG1655 cells per electroporation [16], the identification of only 10-50 recombinants indicates that in our hands the recombination efficiency is approximately 1 in every 3.5 × 10^7 ^cells, 10 times less than reported.

Despite consistently attaining recombinants in MG1655 using this system we have had virtually no success in pathogenic strains. Since the low recombination frequency of the system has been attributed to the inefficient uptake of linear dsDNA fragments during electroporation [4], we determined whether the inefficiency of this system for recombination in pathogenic strains was due to a reduced capacity to uptake DNA by electroporation. Thus, we compared the transformation frequencies of MG1655, O42, CFT073 and O157:H7 Sakai cells when transformed by electroporation with different plasmids. Cells in the exponential phase of growth were transformed by electroporation as previously described [2] with either: pUC18 [17], 2,700 bp (high copy number plasmid), conferring ampicillin resistance; pKD46 [2], 6,300 bp (medium copy number), conferring ampicillin resistance; pACBSR [4], 7,300 bp (medium copy number), conferring chloramphenicol resistance; pRW50 [18], 16,500 bp (low copy number), conferring tetracycline resistance. Cells were then plated onto Lennox broth (LB) agar plates supplemented with appropriate antibiotics, incubated for 20 hours at 37°C and the number of colonies counted. Table 1 shows the transformation frequencies of the pathogenic strains by each plasmid, expressed as a percentage of the transformation frequency of MG1655. It is clear that the transformation frequencies of the pathogenic strains are dramatically lower than for MG1655, particularly for strains CFT073 and O42. Considering that we expect approximately 10-50 recombinants in MG1655, such low electroporation efficiencies could explain why using this technique in pathogenic strains results in minimal success.

**Table 1:** Electroporation efficiencies of *E. coli *strains

	MG1655	O157:H7 Sakai	CFT073	O42
pUC18	100	15	3	0.15
pKD46	100	5	2	0.26
pACBSR	100	2.8	1.5	0
pRW50	100	1.2	1.1	0

pUC18PCR	100	57	15	1

Since the Datsenko and Wanner system relies upon the introduction of PCR generated DNA into cells and not plasmids that have been isolated from an *E. coli *K-12 strain, we re-examined the DNA uptake efficiencies of the strains when transformed with a PCR generated version of the plasmid, pUC18. We reasoned that plasmids isolated from a K-12 strain may be subject to host restriction-modification systems in pathogenic strains, hence, using a PCR-generated pUC18 derivative would not only more closely resemble the conditions used by Datsenko and Wanner, but also allow us to monitor the transformation efficiencies by means of the acquired ampicillin resistance due to pUC18 plasmid uptake. Thus, we amplified pUC18 by PCR and then incubated the reaction with DpnI, which specifically digested the methylated template plasmid and not the PCR generated product. The PCR generated pUC18 plasmid (pUC18PCR) was then transformed into MG1655, CFT073, O157:H7 Sakai and O42 by electroporation. The results (table 1) show that the transformation frequency of the pathogenic strains by pUC18PCR was slightly improved when compared with MG1655, although the overall transformation frequency remains far lower than MG1655. The overall number of MG1655 colonies identified after transformation with pUC18 or pUC18PCR was comparable. Thus, the electroporation step is likely to be the primary reason for the poor efficiency of this system in pathogenic *E. coli *strains. This shortcoming was alleviated somewhat by Murphy and Campellone [15] who developed an improved electroporation based protocol for recombineering in *E. coli *EHEC and EPEC strains. However, we have had mixed success using this protocol, particularly when recombineering in EAEC and UPEC strains, where no increase in recombination frequency was observed.

#### B. Two-plasmid recombineering

The two plasmid gene-gorging method described by Herring and co-workers [4] has an immediate advantage for recombineering in pathogenic strains since the method does not rely upon efficient electroporation as a means of introducing target DNA into the cell. Instead, the target DNA is flanked by recognition sites for the meganuclease I-SceI on a donor plasmid that is transformed into cells along with the recombineering plasmid, pACBSR, which carries I-SceI and the λ-Red genes whose expression is controlled by an arabinose inducible promoter. Induction of I-SceI results in donor plasmid cleavage, generating the linear dsDNA target, which is a substrate for λ-Red gene products. Herring and co-workers disrupted chromosomal genes by introducing amber mutations, using long regions of homology to the chromosome and reported that the recombination frequency for gene gorging was between 1-15%.

To introduce epitope tags at the carboxyl terminus of chromosomal genes and, crucially, to maintain a high-throughput system, we chose to amplify the epitope tag by PCR using oligonucleotide primers that contained 40 bp of homology to the chromosomal gene of interest, rather than sequentially cloning regions of homology to either side of the epitope tag. We reasoned that since short homologous sequences had already been successfully utilised for recombineering by Datsenko and Wanner, [2] this strategy could be adapted for epitope tagging. The amplified DNA product was cloned into pBR322, modified so that the PCR product would be flanked by two recognition sites for I-SceI. The resulting construct was co-transformed, along with pACBSR, into MG1655 cells and gene gorging experiments performed as described by Herring and co-workers [4]. The results of the experiments (not shown) indicated that the recombination efficiency using short regions of homology was very poor; several hundred colonies recovered after gene gorging were screened by PCR and the frequency of recombination was found to be 0.01-0.05%, far less than the 1-15% reported by Herring and co-workers.

To improve the identification rate of recombinants we modified the technique by including a kanamycin cassette adjacent to the epitope tag on the pBR322 based donor plasmid. We reasoned that after *in vivo *digestion of the donor plasmid, the ampicillin cassette carried on pBR322 would be lost and kanamycin resistance would only be maintained if a successful recombination event had occurred. Hence after gene gorging, cells were plated onto LB agar plates containing kanamycin, and the next day colonies were replica plated onto LB plates containing either ampicillin or kanamycin. These colonies were screened for candidates which were kanamycin resistant and ampicillin sensitive, indicative of donor plasmid loss and kanamycin cassette retention as a result of recombination with the chromosome. However, this approach proved to be problematic, since unless the *in vivo *cleavage rate of the donor plasmid by I-SceI approaches 100% efficiency, the ampicillin and kanamycin cassettes are still present on the donor plasmid in the cell, since the plasmid is present in multi-copy, rendering positive selection ineffective. Typically we screened up to 30,000 colonies by replica plating, identifying no more than 5 colonies with the correct phenotype. Taken together these results demonstrate that a more effective technique, that is both rapid and reliable, is required to introduce epitope tags onto the chromosome of pathogenic *E. coli *strains.

### Gene Doctoring

To address this requirement we have developed an enhanced version of the two-plasmid gene gorging system. Our method, termed Gene Doctoring (G-DOC), facilitates the coupling of genes to epitope tags or the deletion of chromosomal genes and increases the rate of identifying recombinants. We have generated a suite of pDOC plasmids which allow for the deletion of chromosomal genes, or the coupling of chromosomal genes to a 6 × His, a 3 × FLAG, a 4 × ProteinA or a GFP tag. The pDOC plasmids carry the *sacB *gene, which allows for the counter-selection of cells still harbouring donor plasmid, thus eliminating the need for screening post recombination. Using this system we routinely identify more than 100 recombinants per experiment in both laboratory and pathogenic *E. coli *strains, using short regions of homology to the chromosome, thus maintaining both a high-throughput and broad-range compatibility system.

### G-DOC plasmids

**Figure 1:**
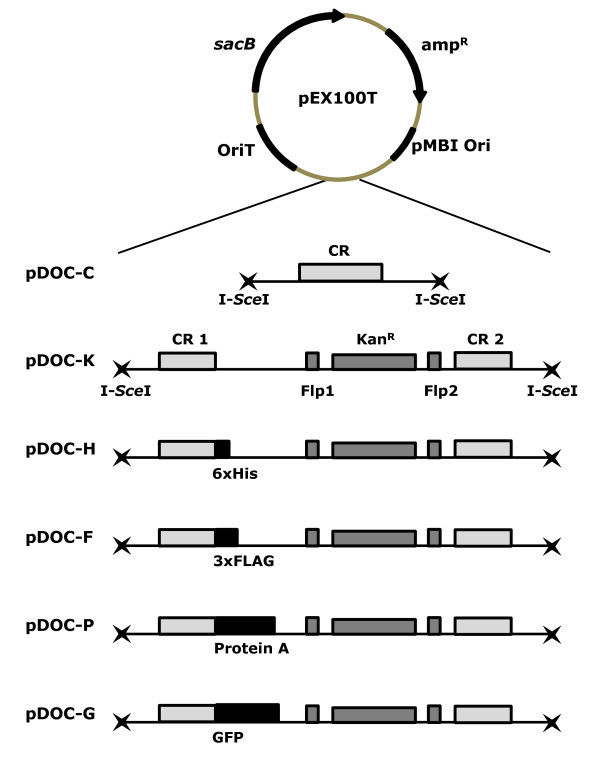
**The pDOC donor plasmids**. Circular representation of the pEX100T plasmid showing the location of the origins of replication, the *sacB *gene and the ampicillin resistance gene. Below is a linear representation of the pDOC plasmid inserts, showing the I-SceI restriction sites, cloning regions (CR, CR1 and CR2), the Flp recognition sites flanking the kanamycin resistance cassette (Kan^R^) and the location of the epitope tags in plasmids pDOC-H, pDOC-F, pDOC-P and pDOC-G.

**Figure 2:**
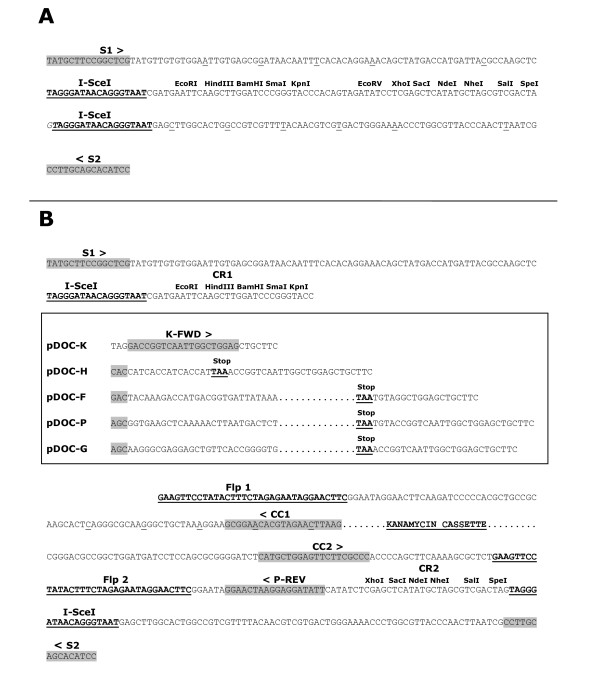
**DNA sequences of the pDOC plasmids**. (A) DNA sequence of pDOC-C insert. The location of sequencing primer annealing sites is indicated (SS1 and SS2). The I-SceI recognition sites are shown flanking the cloning region. (B) DNA sequences of the pDOC-K, pDOC-H, pDOC-F, pDOC-P and pDOC-G inserts. Sequences specific to each plasmid are shown in the open box. The first codon of the epitope tags are highlighted in grey, and the stop codons are indicated. The following primer annealing sites are indicated: SS1 and SS2, used to sequence plasmid derivatives pre-recombination; K-FWD, used for amplifying PCR products from pDOC-K for generating gene deletions; CC1 and CC2, used for generating PCR products in order to confirm recombination; P-REV, used to generate PCR products for cloning into pDOC-C pre-recombination. The Flp recognition sequences are shown (Flp1 and Flp2), flanking the kanamycin cassette. The cloning regions, CR1 and CR2 are shown, adjacent to the I-SceI recognition sites.

The pDOC plasmids are derived from pEX100T, a medium copy number plasmid which carries ampicillin resistance and the *B. subtilis sacB *gene [19]. We have introduced different DNA sequences into the pEX100T I-SceI restriction sites to create a suite of plasmids, schematic diagrams of which are shown in Figure 1. The cloning plasmid, pDOC-C, has a large cloning region (CR) flanked by two I-SceI recognition sites. The DNA sequence of pDOC-C, from 100 bp upstream of the left-hand I-SceI site to 100 bp downstream of the right-hand I-SceI site is shown in Figure 2, panel A. The template plasmid, pDOC-K, carries a kanamycin resistance cassette flanked by Flp recombinase recognition sites (Flp1 and Flp2). On either side of this region are 2 cloning regions (CR1 and CR2). The other template plasmids, pDOC-H, pDOC-F, pDOC-P and pDOC-G are derivatives of pDOC-K that have the coding sequence for a 6 × His, 3 × FLAG, 4 × Protein A and GFP tag respectively, immediately downstream of CR1. Figure 2; panel B, shows the DNA sequence common to all of the pDOC template plasmids, from 100 bp upstream of the left-hand I-SceI site to 100 bp downstream of the right-hand I-SceI site. The template plasmids differ between the CR1 and FLP1 sequences: this region is outlined by an open box in the figure. The DNA sequence proceeds through CR1, along the respective DNA sequence for each plasmid within the open box, and into the FLP1 sequence below. The plasmid pDOC-K has 30 bp of DNA sequence prior to FLP1. The plasmid pDOC-H has the coding sequence for the 6 × His tag and a stop codon followed by a short DNA sequence leading into the FLP1 site. The first 10 codons of the 3 × FLAG, ProteinA and GFP tags are shown, followed by the stop codon and short DNA sequences leading into FLP1 site. Other features indicated on the DNA sequences of the pDOC plasmids in Figure 2 are described in the G-DOC recombineering protocol below. The full DNA sequence of each pDOC plasmid is provided in Additional file 1 and is also available from GenBank, accession numbers GQ88494-GQ889498.

### G-DOC recombineering protocol

**Figure 3:**
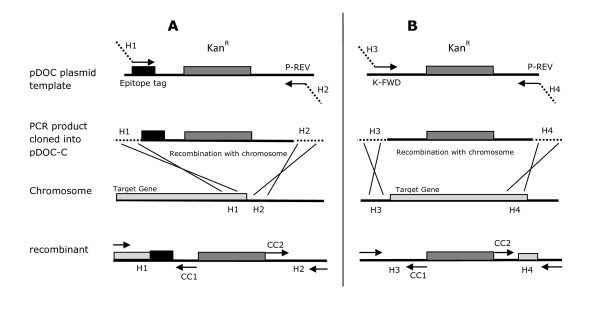
**Schematic of pDOC based recombination**. PCR products are generated for gene coupling (A) or for gene deleting (B) and cloned into pDOC-C. Homologous regions (H1-4) on the PCR product recombine with the target gene on the chromosome. Recombinant clones are then checked by PCR using primers annealing to the CC1 and CC2 sequences, and sequences adjacent to the homology regions.

For generating gene:epitope tag fusions, the epitope tag and kanamycin cassette are amplified by PCR, using the relevant pDOC plasmid as a template. A schematic outline of the cloning strategy for generating gene:epitope tag fusions is shown in Figure 3, panel A. The clockwise primer used for the PCR amplification is designed so that it contains between 25-50 bp of homology to the 3' end of the target gene (H1), not including the stop codon, followed by 20 bp of sequence which anneals to the epitope tag sequence on the pDOC plasmid. This should be designed so that, after recombination with the target gene on the chromosome, the gene will be in frame with the coding sequence of the epitope tag. The downstream primer is designed so that it contains 25-50 bps of homology to the DNA sequence immediately downstream of the target gene (H2) and the primer sequence P-REV. The two primers are also designed with a restriction site at the 5' end, so that, after amplification by PCR, the DNA product can be cloned into the cloning region of pDOC-C, between the two I-SceI sites.

For generating gene deletions, the kanamycin cassette from pDOC-K, is amplified by PCR. A schematic outline of the cloning strategy for generating gene deletions is shown in Figure 3, panel B. The clockwise primer used for the PCR amplification is designed so that it contains between 25-50 bp of homology to the DNA immediately upstream of the start of the gene (H3), followed by 20 bp of sequence which anneals to the K-FWD sequence on pDOC-K. The anti-clockwise primer is designed so that it contains 25-50 bps of homology to the 3' end of the gene (H4) and the primer sequence P-REV. Again, the two primers are designed with 5' restriction sites for cloning the DNA product into pDOC-C.

Alternatively, when longer regions of homology to the chromosome are required, sequential cloning steps can be performed, utilising the multiple cloning sites to introduce long regions of chromosomal homology upstream and downstream of the kanamycin cassette and epitope tag. In this case we recommend sequencing the cloned homology regions, post cloning and before recombineering, using priming sequences S1 and S2 (highlighted in Figure 2: primers D58794 and D58793).

**Figure 4:**
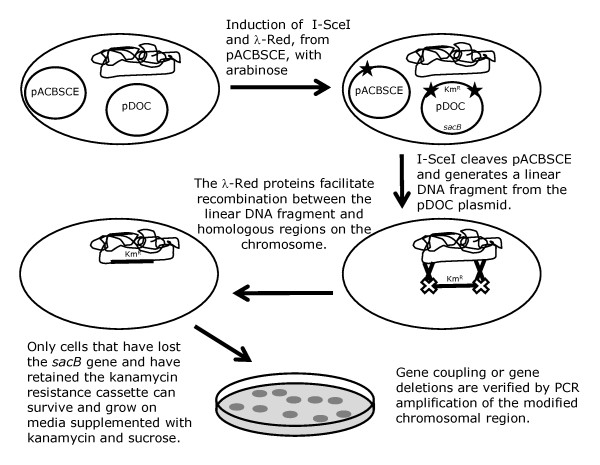
**G-DOC recombineering**. The pDOC donor plasmid and the recombineering plasmid pACBSCE are co-transformed into the recipient strain. Arabinose induction promotes expression of the λ-Red gene products and I-SceI. I-SceI generates a linear DNA fragment form the donor plasmid that is a substrate for recombination with the chromosome mediated by the λ-Red system. Recombinants are selected by the ability to survive and grow on LB supplemented with kanamycin and sucrose.

The next step is to transform the pDOC donor plasmid into the recipient strain with the recombineering plasmid, which expresses I-SceI and the λ-Red gene products. A schematic protocol, outlining the key steps in generating recombinants is shown in Figure 4. We have modified the recombineering plasmid, pACBSR, used by Herring and co-workers [4] by introducing an I-SceI recognition site adjacent to the replication origin of the plasmid: we have called this plasmid pACBSCE. Upon arabinose induction, a burst of I-SceI and λ-Red expression occurs; I-SceI cleaves the donor plasmid resulting in generation of the substrate for λ-Red mediated recombination. In addition, I-SceI also cleaves the pACBSCE recombineering plasmid, resulting in loss of plasmid and loss of λ-Red expression, thus avoiding prolonged λ-Red activity, which can result in unwanted chromosomal modification [13-15]. Recombination occurs between homologous regions on the linear DNA substrate and the chromosome, transferring the kanamycin cassette, and in the case of gene:coupling, the epitope tag, onto the chromosome (Figures 3 and 4). Recombinant clones are selected for by growing cells on LB agar plates containing kanamycin and sucrose: only true recombinants, which have lost the *sacB *gene due to donor plasmid loss and have retained the kanamycin cassette due to recombination, are able to survive and grow on this medium. Examination of recombinants, to ensure that the correct chromosomal modification has been generated, is achieved by amplifying the target region by PCR, using primers that anneal adjacent to the homology regions (H1-4 in figure 3) and chromosomal check priming sequences CC1 and CC2 (Figure 2, panel B and Figure 3). Once recombination has been confirmed, the kanamycin cassette can be excised from the chromosome using the Flp recombinase sites, as described previously. [2]

### G-DOC validation

To test our system we designed oligonucleotides to couple the *rsd *and *yacL *genes to the 3 × FLAG epitope tag. One primer contained homology to the first 7 codons of the 3 × FLAG sequence, 40 bp of homology to the 5' end of the target gene, excluding the stop codon, and an EcoRI restriction site (primer D61350 for *rsd *and D61352 for *yacL*). The second primer contained homology to the P-REV sequence, 40 bp of homology to the chromosome, immediately downstream of the target gene primer and a KpnI restriction site (D61351 for *rsd *and D61353 for *yacL*). DNA fragments generated by PCR using pDOC-F as a template were cloned into pDOC-C, which was subsequently co-transformed with pACBSCE into K-12 MG1655, EHEC O157:H7 Sakai and UPEC CFT073 cells. The Gene Doctoring protocol was followed and the results are reported in table 2. For both genes, in all three strains, a large number of colonies were identified with a kanamycin resistant, sucrose insensitive phenotype. After PCR analysis of the relevant chromosomal region (using primer pairs D57786 (CC1) and D61354, and D57785 (CC2) and D61355 for *rsd *and D57786 and D61356, and D57785 and D61357 for *yacL*) the vast majority of candidates were found to be true recombinants and in each case, more than 90% were sensitive to both ampicillin and chloramphenicol, indicating loss of both pDOC donor and pACBSCE plasmids. Where a candidate was found to have the wild-type form of the gene after PCR verification, we assumed that the kanamycin cassette had inserted into a different part of the chromosome, since we were unable to isolate any donor plasmid DNA from cells using standard plasmid isolation techniques. Hence, for each gene, in each strain, more than 150 recombinants were identified that had the correct chromosomal modification and were free of the recombineering plasmid pACBSCE.

**Table 2:** Comparison of recombination efficiency of *E. coli *strains

	Kan^R ^Suc^I^ (A)	recombinants	Plasmid free recombinants (B)	% plasmid free recombinants (B/A)
*rsd*				

MG1655	249	248	232	93
O157:H7 Sakai	193	193	184	95
CFT073	174	170	156	90

*yacL*				

MG1655	287	286	258	90
O157:H7 Sakai	218	218	209	96
CFT073	209	205	192	92

To test the effectiveness of recombination using our recombineering plasmid pACBSCE, compared with the recombineering plasmid pACBSR, used by Herring and co-workers [4] we repeated the gene coupling analysis of the *rsd *gene. The results in table 3 show that more kanamycin resistant, sucrose insensitive recombinants were identified in each strain when pACBSR was used as the recombineering plasmid, with a comparable percentage being free of pDOC donor plasmid, when compared to using pACBSCE as the recombineering plasmid. However, very few candidates had lost the recombineering plasmid, and in strain CFT073, all of the recombinant candidates still carried pACBSR, thus exposing cells to the potential effects of excess of λ-Red expression and requiring additional steps to cure cells of the plasmid [4,13-15].

**Table 3:** Comparison of recombination efficiency using recombineering plasmids pACBSCE and pACBSR

	Kan^R^+Suc^I^		% free of pDOC donor plasmid		% free of recombineering plasmid	
**Recombineering plasmid**	**pACBSCE**	**pACBSR**	**pACBSCE**	**pACBSR**	**pACBSCE**	**pACBSR**

MG1655	249	320	94	93	97	9
O157:H7 Sakai	193	308	97	99	97	3
CFT073	174	234	91	93	99	0

We have further validated the system by making several gene deletions in both laboratory and pathogenic strains. DNA fragments, generated by PCR amplification, using pDOC-K as a template were cloned into pDOC-C, and the resulting donor plasmids used for gene doctoring. To date we have made deletions of the *rpoS, fur, flhDC *and *soxS *genes in MG1655, O157:H7 Sakai, CFT073 and H10407 strains (data not shown).

### Functionality of the epitope tags

**Figure 5:**
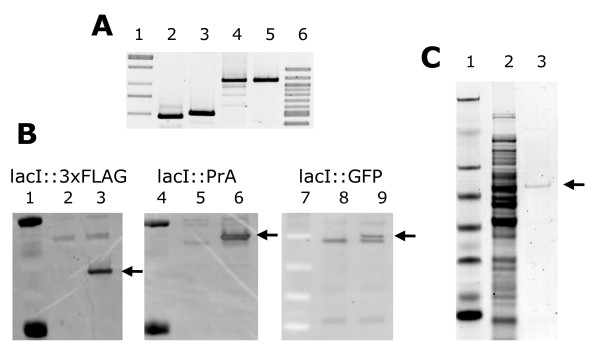
**Verification and functionality of chromosomal *lacI::tag *fusions**. (A) Ethidum bromide stained agarose gel showing DNA amplified by PCR from the *lacI *fusion strains. Lanes 1 and 6 are DNA markers, lanes 2, 3, 4 + 5 show DNA derived from *lacI::6 × his, lacI::3 × FLAG*, *lacI::ProteinA *and *lacI::GFP *respectively. (B) Western blot analysis of tagged strains. Lanes 1, 4 and 7 show protein standards. Lanes 2, 5 and 8 show wild-type MG1655. Lanes 3, 6 and 9 show the tagged strains. Lanes 1, 2 + 3 were probed with antibody specific to the FLAG tag, lanes 4, 5 +6 were probed with antibody specific to ProteinA and lanes 7, 8 + 9 were probed with antibody specific to GFP. (C) SDS-PAGE analysis of the affinity-isolation of LacI::6 × His. Proteins were stained with Coomassie blue. Lane 1 shows protein standards, lane 2, whole cell extract, lane 3, LacI::6 × His affinity-isolate.

To examine the functionality of the epitope tags we coupled each to the Lac repressor protein in MG1655. The experimental details and primer design for each recombination experiment are given in the methods section. For each epitope tag we identified more than 200 candidates that were kanamycin resistant, sucrose insensitive. After verification by PCR amplification and DNA sequencing of the chromosomal region (Figure 5; panel A), we tested the functionality of the epitope tags. The LacI::3 × FLAG, LacI::4 × ProteinA and LacI::GFP fusion proteins were analyzed by Western blotting. Whole cell extracts were separated by SDS-PAGE and proteins transferred to nitrocellulose membranes, which were then probed with primary antibodies specific to the tag. The membranes were then washed and probed with secondary antibodies conjugated to horse-radish peroxidase. Figure 5; panel B, shows an image of the membranes after exposure to X-ray film; the fusion proteins are indicated. In a recent study we validated the functionality of the LacI::3 × FLAG fusion protein by isolating DNA fragments carrying LacI binding sites from cells [20]. We also confirmed the fluorescence of the LacI::GFP fusion protein, in whole cells using fluorescent microscopy (data not shown). Finally, we tested the integrity of the 6 × His fusion proteins by isolating the protein fusion by affinity purification using nickel agarose affinity media (Qiagen). Purified proteins were analysed by SDS-PAGE. Figure 5; panel C, shows a scanned image of the SDS-PAGE gel on which the fusion protein is highlighted.

## Conclusion

We have developed a version of the two-plasmid recombineering system for generating chromosomal modifications in *E. coli *strains which, we have termed Gene Doctoring. This method relies on homologous recombination, mediated by the λ-Red genes, of a linear DNA fragment that is supplied *in vivo *by restriction of a pDOC donor plasmid by I-SceI endonuclease. The identification of recombinants is highly efficient and reproducible, since counter-selection using the *sacB *gene identifies true recombinants. This eliminates the requirement for screening large numbers of candidates by PCR, which is both costly and time consuming. In addition, we have made a modified recombineering plasmid, pACBSCE, which carries a DNA recognition site for I-SceI in the origin of replication, meaning that recombinants are not over-exposed to the potential mutagenic side-effects of the λ-Red gene products.

The gene doctoring system is principally effective for recombineering in different pathogenic *E. coli *strains, which as we have demonstrated, are not particularly amenable to chromosomal modification using existing systems. This system is designed to facilitate the coupling of genes to epitope tags, though the deletion of genes can also be readily achieved. We have demonstrated the versatility of Gene Doctoring by deleting genes in both laboratory and pathogenic *E. coli *strains, in addition to coupling several genes to epitope tags, and we have confirmed the functionality of epitope tagged fusion proteins using biochemical methods. We believe that the gene doctoring system can be transferable to other bacteria, in which the pDOC and pACBSCE plasmids are stable and will replicate.

## Methods

### Strains

The *E. coli *strains used in this study were MG1655 K-12 strain [21], O157:H7 Sakai EHEC strain (derivative in which the *stx1 *and *stx2 *genes were deleted by M. D. Goldberg, University of Birmingham, UK) [8], CFT073 UPEC strain [9] O42 EAEC [10] and H10407 ETEC strains [11] (from Ian R. Henderson, University of Birmingham, UK; Sequenced by the Sanger Institute: unpublished).

### Primers

The primers used in this study are listed in table 4.

**Table 4:** Primers used in this study

Name	Sequence 5' - 3'
D55908	ATATGGTACCGACTACAAAGACCATGACGG
D55909	TGACGAGCTCTATACTTATAGGAGGAATCAAGG
D57236	CTAGGGATAACAGGGTAATCGATGAATTCAAGCTTGGATCCCGGGTAC
D57237	CCGGGATCCAAGCTTGAATTCATCGATTACCCTGTTATCCCTAGACGT
D57238	TCGAGCTCATATGCTAGCGTCGACTAGTAGGGATAACAGGGTAATGCA
D57239	TTACCCTGTTATCCCTACTAGTCGACGCTAGCATATGAGC
D57436	GATCGGTACCTAGGACCGGTCAATTGACGCGTCTTAAGGGAATTGCCAGCTGGGGCGC
D57437	GTCTCTTGATCAGATCTTGATCC
D57584	AATGGTACCAGCGGTGAAGCTC
D57585	GAATACCGGTACATTACCCGGCATCGTC
D57785	GAATCTTAAGTTCTACGTGTTCCGC
D57786	CATGCTGGAGTTCTTCGCCC
D58793	GGATGTGCTGCAAGG
D58794	TATGCTTCCGGCTCG
D59400	GCTGAATTCCAAACAGGATTTTCGCCTGC
D59401	GTCGGTACCCTGCCCGCTTTCCAGTC
D59402	GTCCTCGAGGTTGATGAAAGCTGGCTAC
D59403	GTAGCTAGCTCCGCCACATATCCTG
D59990	TAGGGTACCAGCAAGGGCGAGGAGC
D59991	TAGACCGGTTTACTTGTACAGCTCGTCC
D60111	CCACAGTAGATATCC
D60112	TCGAGGATATCTACTGTGGGTAC
D60113	CAGGTTTCCCGACTGGAAAGCGGGCAGCCGGGTACCCACCATCAC
D60114	AGCTAACTCACATTAATTGCGTTGCGCAATATCCTCCTTAGTTCC
D61347	CGTTGGTGCGGATATCTCGG
D61350	GACGAATTCTCTGCTGGTGCTTGACGCCGCCCGCGTCAAACATCCTGCTTACAAAGACCATGACGGTGAT
D61351	CAGGGTACCTGGGGCATTGAATGTAAATTACGCGTTAACAGCGCAGAACAATATCCTCCTTAGTTCC
D61352	GACGAATTCGCAGGTCGTGGCGGCTTACCGCAATTTCGTGCAGCAGAAGTACAAAGACCATGACGGTGAT
D61353	CAGGGTACCTAACGGCTCTGGCGGAGCTCCCAGGCTCCGCCAGATTTATAATATCCTCCTTAGTTCC
D61354	TACCTTGAGTTTCAACAGG
D61355	GATCCCGAATAAACGGTC
D61356	CAATCAACTGGAATTCGC
D61357	CGGAAGACTTAACCGACAGC
D61358	TAGGGATAACAGGGTAATGAGCGGAAATGGCTTACGAAC
D61359	GCCGCAGTCGAACGACCGAG
D61360	GTTGGCACTGATGAGGGTGT
D66425	CTCGAATTCGTAATCATGGTCATAGCTGTTTCCTG
D66426	CAGGAAACAGCTATGACCATGATTACGAATTCGAG

### Generation of pUC18PCR

The plasmid pUC18PCR was generated by PCR using primers D66425 and D66426, with pUC18 used as a template in the reaction. The two primers were designed so that 35 bp complementary overhangs remained at each 5' terminus of the amplicon, facilitating plasmid re-circularisation pre-transformation. The restriction enzyme DpnI was then added to the reaction in order to digest specifically the methylated pUC18 template plasmid, leaving only the PCR generated pUC18PCR intact.

### Construction of the pDOC plasmids

The pDOC series of plasmids are based on the plasmid pEX100T [19]. A schematic representation of the plasmids is shown in Figure 2 and the entire DNA sequence of each plasmid is provided in Additional file 1 and also on GenBank; nos.. The plasmid pDOC-F was constructed by amplification of the 3 × FLAG DNA sequence, Flp recombinase target sites (Flp1 and Flp2) and the kanamycin cassette from the plasmid pSUB11 [22], flanked by KpnI and XhoI restriction sites, using the primers D55908 and D55909. This amplicon was cloned into the pGEM-T Easy cloning vector (Promega). The resulting plasmid was then restricted with AatII and KpnI, and ligated with annealed complementary oligonucleotides (D57236 and D57237), designed to introduce an I-SceI site and a multi-cloning region (CR1). This plasmid was then restricted with XhoI and NsiI and ligated with annealed complementary oligonucleotides (D57238 and D57239), designed to introduce a second I-SceI site and a multi-cloning region (CR2). The resulting plasmid was restricted with I-SceI, and the fragment cloned into the I-SceI sites of the plasmid pEX100T.

To facilitate further plasmid construction, the 3 × FLAG DNA sequence was removed from pDOC-F, and an AgeI restriction site introduced immediately downstream of the KpnI site. This was achieved by PCR amplification of the DNA immediately downstream of the 3 × FLAG DNA sequence, flanked by KpnI and BglII restriction sites using primers D57436 and D57437. This fragment was then cloned into the unique KpnI and BglII sites of pDOC-F, effectively removing the 3 × FLAG DNA sequence. This new plasmid, pDOC-K, retained the Flp recombination target sites and the kanamycin cassette.

The plasmid pDOC-K was restricted with KpnI and AgeI, and KpnI-AgeI flanked DNA harboring a 6 × His coding sequence, the coding sequence of GFP (from Invitrogen Emerald Green GFP gateway vector - V355-20; amplified with primers D59990 and D59991) or the coding sequence of ProteinA [23] (amplified using primers D57584 and D57585), were ligated. This resulted in the generation of the plasmids pDOC-H, pDOC-G and pDOC-P respectively.

The plasmid pDOC-C was created by removing the Flp recombinase sites and the kanamycin cassette from pDOC-K, by digestion with KpnI-XhoI, and ligation with annealed complementary oligonucleotides that introduced a unique EcoRV site (D60111 and D60112).

### Construction of pACBSCE

Plasmid pACBSR [4] was used as a template in PCR to create pACBSCE, using the primers D61358 and D61359, which anneal adjacent to the origin of replication. D61358 contains the recognition sequence for I-SceI at the 5' end. The resulting linearised plasmid, carrying an I-SceI recognition site, was self-ligated using a Quick Ligation Kit (NEB). Circularized plasmid was transformed into TOP10 cells (Invitrogen) and plated onto LB agar plates supplemented with chloramphenicol (35 μg/ml). The plasmid was then checked by digestion with I-SceI enzyme (N.E.B.), and sequenced using primer D61360.

### Gene-doctoring protocol

Electrocompetent *E. coli *cells were transformed with the recombineering plasmid, pACBSCE, and a pDOC donor plasmid derivative and spread onto LB agar plates containing 35 μg/ml chloramphenicol (for pACBSCE) and 200 μg/ml ampicillin and 50 μg/ml kanamycin (for pDOC derivatives)(LB_CAK _agar plates). Colonies were routinely tested for maintenance of the *sacB *gene on the pDOC donor plasmid, by patching onto LB_CAK _agar plates and LB_CAK _agar plates supplemented with 5% sucrose: colonies containing a functional *sacB *gene will be unable to grow on plates supplemented with 5% sucrose. A single fresh sucrose sensitive colony was inoculated into 1 ml of LB_CAK_, supplemented with 0.5% Glucose, which was added to prevent leaky expression of the λ-Red and *I-SceI *genes from pACBSCE. Cultures were incubated with shaking at 37°C for 2 hours. Cells were harvested by centrifugation and re-suspended in 1 ml LB containing 0.5% L-arabinose which was added to induce expression of the λ-Red and *I-SceI *genes from pACBSCE. Note that antibiotics were omitted from the growth medium at this stage. The culture was incubated at 37°C, with vigorous shaking until turbid (approximately 4-5 hours). Dilutions of the culture were then plated on to LB agar plates containing 50 μg/ml kanamycin and 5% sucrose and incubated overnight at 30°C. Kanamycin resistant, sucrose insensitive recombinants were checked for donor plasmid loss by patching onto LB agar plates containing 200 μg/ml ampicillin and pACBSCE plasmid loss by patching onto LB agar plates containing 35 μg/ml chloramphenicol. Recombination was confirmed by PCR and sequencing, using oligonucleotide primers homologous to chromosomal DNA flanking the modified region (sequencing provided by the Birmingham Functional Genomics laboratory). Note: in addition, dilutions of the culture were routinely plated onto LB agar plates and LB agar plates supplemented with 200 μg/ml of ampicillin, to quantify the amount of donor plasmid digestion by I-SceI and LB agar plates and LB agar plates supplemented with 35 μg/ml chloramphenicol, to quantify pACBSCE digestion by I-SceI.

### Construction of pDOC derivatives for generating lacI gene fusions

Four different *lacI *gene fusions were constructed in MG1655, producing the following recombinant proteins; LacI::6 × His, LacI::3 × FLAG, LacI::4 × ProteinA and LacI::GFP. For the LacI::6 × His construct, two primers were designed to amplify the 6 × his coding region and the kanamycin cassette from pDOC-H: the first primer, D60113, included 27 bp of homology to the C-terminus of *lacI*, excluding the stop codon, and 18 bp homology to pDOC-H and was designed so that the 6 × his sequence was in frame with the *lacI *coding sequence. The second primer, D60114 included 27 bp of homology to the region immediately downstream of *lacI*, and homology to the P-REV annealing sequence. These primers were used to amplify the kanamycin resistance cassette, using pDOC-H as a template, and a proof-reading thermostable DNA polymerase that produces a blunt-ended amplicon. The resulting fragment was blunt end ligated into the EcoRV site of pDOC-C. The cloned region was sequenced using primers D58793 and D58794, which anneal to the S1 and S2 sites (Figure 2) in the pDOC-C plasmid. The resulting plasmid was then used to tag the chromosomal *lacI *gene in *E. coli *strain MG1655 by gene doctoring. Recombinants were checked by PCR and sequencing using primers D61347, which anneals within the *lacI *gene, and D57785, which anneals to the CC1 sequence shown in Figure 2.

The *lacI*::3 × FLAG, *lacI*::4 × ProteinA and *lacI*::GFP gene fusions were made using longer regions of homology to the chromosome, cloned directly into the pDOC-F, pDOC-P and pDOC-G cloning regions. The C-terminal 200 bp of the *lacI *gene, excluding the stop codon, was amplified by PCR using primers D59400 and D59401, and cloned into CR1 of the appropriate tagging vector, on a EcoRI:KpnI fragment, arranged so that the coding sequence of the gene was in frame with the epitope tag. Next, a 200 bp region of the *lacZ *gene (codons 130-205) was amplified by PCR using primers D59402 and D59403 and cloned into CR2 of the appropriate tagging vector, on a XhoI:NheI fragment. The resulting plasmids were then used to tag the chromosomal *lacI *gene in *E. coli *strain MG1655 by gene doctoring. Recombinants were checked by PCR and DNA sequencing as before.

### Western blotting

Cells of strain MG1655lacI::3 × FLAG, MG1655lacI::4 × ProteinA and MG1655lacI::gfp were grown in LB medium at 37°C to an OD_650 _of 1.2. Samples were taken and cell extracts were separated on a SDS-PAGE gel. Proteins were then transferred to a nitrocellulose membrane, which was probed with antibodies specific for the FLAG peptide (Sigma), ProteinA (Sigma) or GFP (Roche). The membranes were then incubated with HRP-labeled anti-mouse IgG (Sigma), and binding of antibody visualized by scanning with a Syngene Gene Genius Bioimaging System.

### Affinity isolation of LacI::6 × His

A 100 ml culture of strain MG1655lacI::6 × his was grown in LB medium at 37°C to an OD_650 _of 1.2. Cells were harvested and re-suspended in 4 mls of lysis buffer (10 mM Tris, 100 mM NaCl, 10% Glycerol). Lysozyme was added to a final concentration of 400 μg/ml, and the mixture incubated on ice for 30 minutes, with regular mixing. After lysozyme treatment, the lysate was cleared by centrifugation and the supernatant incubated with 200 μl of NTA-Ni-agarose beads (Qiagen), on ice for 30 minutes. The supernatant was then removed, and the beads washed with 1 ml of wash buffer (10 mM Tris, 100 mM NaCl, 10% Glycerol, 10 mM Imidazole). LacI::6 × His was then eluted from the beads with 100 μl of elution buffer (10 mM Tris, 100 mM NaCl, 10% Glycerol, 250 mM Imidazole).

## Authors' contributions

DJL constructed the pDOC plasmids, designed the protocol, performed the experiments and co-wrote the manuscript. LEHB constructed and tested the pACBSCE recombineering plasmid and assisted in protocol design. KH constructed the *rpoS, fur, flhDC *and *soxS *genes in the *E. coli *MG1655, O157:H7 Sakai, CFT073 and H10407 strains, assisted in protocol design and co-wrote the manuscript. MJP, CWP and SJWB provided supervision and assisted in editing of the final manuscript. JLH assisted in plasmid and protocol design, provided technical advice and supervision and co-wrote the manuscript. All of the authors have read and approved this manuscript.

## Supplementary Material

Additional file 1 **Annotated sequence of the pDOC plasmids**. The file contains the DNA sequence of each pDOC plasmid with annotation of open reading frames, multi-cloning sites and primer binding sites. Click here for file
