# Supplementary material for: Gene doctoring: a method for recombineering in laboratory and pathogenic Escherichia coli strains
Source: BMC Microbiol. 2009 Dec 9;9:252. doi: 10.1186/1471-2180-9-252 (PMC2796669; doi:10.1186/1471-2180-9-252)
Supplement: Additional file 1 — Annotated sequence of the pDOC plasmids. The file contains the DNA sequence of each pDOC plasmid with annotation of open reading frames, multi-cloning sites and primer binding sites. [file 1471-2180-9-252-S1.DOC]

**Additional file 1**

This file contains the annotated sequences of the pDOC donor plasmids.

For pDOC-C the coding sequence of the *bla* and *sacB* gene are shown. Also highlighted are the I-SceI recognition sites and the multi-cloning region.

For pDOC-K the coding sequence of the *bla* and *sacB* genes are shown as well as the coding sequence for the kanamycin rsistance gene (*kan*)

For pDOC-H, -F, -P and –G the coding sequence of the *bla* and *sacB* genes are shown as well as the coding sequence for the kanamycin rsistance gene (*kan*) and the plasmid specific epitope tag. Also highlighted are the multi-cloning regions, the I-SceI recognition sites and the FLP recombinase recognition sites.

The DNA sequence for each plasmid is also available from GenBank – nos. GQ889494-GQ889498

**Page 1 – pDOC-C: Page 7 - pDOC-K: Page 14 – pDOC-H:**

**Page 21 – pDOC-F: Page 28 – pDOC-P: Page 35 – pDOC-G.**

**pDOC-C**

0  GACGAAAGGGCCTCGTGATACGCCTATTTTTATAGGTTAATGTCATGATAATAATGGTTT

   60  CTTAGACGTCAGGTGGCACTTTTCGGGGAAATGTGCGCGGAACCCCTATTTGTTTATTTT

  120  TCTAAATACATTCAAATATGTATCCGCTCATGAGACAATAACCCTGATAAATGCTTCAAT

  180  AATATTGAAAAAGGAAGAGTATGAGTATTCAACATTTCCGTGTCGCCCTTATTCCCTTTT

    3                      M  S  I  Q  H  F  R  V  A  L  I  P  F  F

**bla >>**

  240  TTGCGGCATTTTGCCTTCCTGTTTTTGCTCACCCAGAAACGCTGGTGAAAGTAAAAGATG

    3    A  A  F  C  L  P  V  F  A  H  P  E  T  L  V  K  V  K  D  A

  300  CTGAAGATCAGTTGGGTGCACGAGTGGGTTACATCGAACTGGATCTCAACAGCGGTAAGA

    3    E  D  Q  L  G  A  R  V  G  Y  I  E  L  D  L  N  S  G  K  I

  360  TCCTTGAGAGTTTTCGCCCCGAAGAACGTTTTCCAATGATGAGCACTTTTAAAGTTCTGC

    3    L  E  S  F  R  P  E  E  R  F  P  M  M  S  T  F  K  V  L  L

  420  TATGTGGCGCGGTATTATCCCGTATTGACGCCGGGCAAGAGCAACTCGGTCGCCGCATAC

    3    C  G  A  V  L  S  R  I  D  A  G  Q  E  Q  L  G  R  R  I  H

  480  ACTATTCTCAGAATGACTTGGTTGAGTACTCACCAGTCACAGAAAAGCATCTTACGGATG

    3    Y  S  Q  N  D  L  V  E  Y  S  P  V  T  E  K  H  L  T  D  G

  540  GCATGACAGTAAGAGAATTATGCAGTGCTGCCATAACCATGAGTGATAACACTGCGGCCA

    3    M  T  V  R  E  L  C  S  A  A  I  T  M  S  D  N  T  A  A  N

  600  ACTTACTTCTGACAACGATCGGAGGACCGAAGGAGCTAACCGCTTTTTTGCACAACATGG

    3    L  L  L  T  T  I  G  G  P  K  E  L  T  A  F  L  H  N  M  G

  660  GGGATCATGTAACTCGCCTTGATCGTTGGGAACCGGAGCTGAATGAAGCCATACCAAACG

    3    D  H  V  T  R  L  D  R  W  E  P  E  L  N  E  A  I  P  N  D

  720  ACGAGCGTGACACCACGATGCCTGTAGCAATGGCAACAACGTTGCGCAAACTATTAACTG

    3    E  R  D  T  T  M  P  V  A  M  A  T  T  L  R  K  L  L  T  G

  780  GCGAACTACTTACTCTAGCTTCCCGGCAACAATTAATAGACTGGATGGAGGCGGATAAAG

    3    E  L  L  T  L  A  S  R  Q  Q  L  I  D  W  M  E  A  D  K  V

  840  TTGCAGGACCACTTCTGCGCTCGGCCCTTCCGGCTGGCTGGTTTATTGCTGATAAATCTG

    3    A  G  P  L  L  R  S  A  L  P  A  G  W  F  I  A  D  K  S  G

  900  GAGCCGGTGAGCGTGGGTCTCGCGGTATCATTGCAGCACTGGGGCCAGATGGTAAGCCCT

    3    A  G  E  R  G  S  R  G  I  I  A  A  L  G  P  D  G  K  P  S

  960  CCCGTATCGTAGTTATCTACACGACGGGGAGTCAGGCAACTATGGATGAACGAAATAGAC

    3    R  I  V  V  I  Y  T  T  G  S  Q  A  T  M  D  E  R  N  R  Q

 1020  AGATCGCTGAGATAGGTGCCTCACTGATTAAGCATTGGTAACTGTCAGACCAAGTTTACT

    3    I  A  E  I  G  A  S  L  I  K  H  W

 1080  CATATATACTTTAGATTGATTTAAAACTTCATTTTTAATTTAAAAGGATCTAGGTGAAGA

 1140  TCCTTTTTGATAATCTCATGACCAAAATCCCTTAACGTGAGTTTTCGTTCCACTGAGCGT

 1200  CAGACCCCGTAGAAAAGATCAAAGGATCTTCTTGAGATCCTTTTTTTCTGCGCGTAATCT

 1260  GCTGCTTGCAAACAAAAAAACCACCGCTACCAGCGGTGGTTTGTTTGCCGGATCAAGAGC

 1320  TACCAACTCTTTTTCCGAAGGTAACTGGCTTCAGCAGAGCGCAGATACCAAATACTGTCC

 1380  TTCTAGTGTAGCCGTAGTTAGGCCACCACTTCAAGAACTCTGTAGCACCGCCTACATACC

 1440  TCGCTCTGCTAATCCTGTTACCAGTGGCTGCTGCCAGTGGCGATAAGTCGTGTCTTACCG

 1500  GGTTGGACTCAAGACGATAGTTACCGGATAAGGCGCAGCGGTCGGGCTGAACGGGGGGTT

 1560  CGTGCACACAGCCCAGCTTGGAGCGAACGACCTACACCGAACTGAGATACCTACAGCGTG

 1620  AGCTATGAGAAAGCGCCACGCTTCCCGAAGGGAGAAAGGCGGACAGGTATCCGGTAAGCG

 1680  GCAGGGTCGGAACAGGAGAGCGCACGAGGGAGCTTCCAGGGGGAAACGCCTGGTATCTTT

 1740  ATAGTCCTGTCGGGTTTCGCCACCTCTGACTTGAGCGTCGATTTTTGTGATGCTCGTCAG

 1800  GGGGGCGGAGCCTATGGAAAAACGCCAGCAACGCGGCCTTTTTACGGTTCCTGGCCTTTT

 1860  GCTGGCCTTTTGCTCACATGTTCTTTCCTGCGTTATCCCCTGATTCTGTGGATAACCGTA

 1920  TTACCGCCTTTGAGTGAGCTGATACCGCTCGCCGCAGCCGAACGACCGAGCGCAGCGAGT

 1980  CAGTGAGCGAGGAAGCGGAAGAGCGCCCAATACGCAAACCGCCTCTCCCCGCGCGTTGGC

 2040  CGATTCATTAATGCAGCTGGCACGACAGGTTTCCCGACTGGAAAGCGGGCAGTGAGCGCA

 2100  ACGCAATTAATGTGAGTTAGCTCACTCATTAGGCACCCCAGGCTTTACACTTTATGCTTC

 2160  CGGCTCGTATGTTGTGTGGAATTGTGAGCGGATAACAATTTCACACAGGAAACAGCTATG

*HindIII*

                                SceI             **EcoRI**       BamHI

 2220  ACCATGATTACGCCAAGCTCTAGGGATAACAGGGTAATCGAT**GAATTC***AAGCTT*GGAT**CC**

**XmaI**                  XhoI      **NheI**      *SpeI*

**SmaI** KpnI       **EcoRV**   **SacI** *NdeI*      SalI         SceI

 2280  **CGGG**TACCCACAGTA**GATATC**CTC**GAGCT*C****ATAT****G*CTAGC**GTCG*ACTAGT*AGGGATAACA

 2340  GGGTAATGAGCTTGGCACTGGCCGTCGTTTTACAACGTCGTGACTGGGAAAACCCTGGCG

 2400  TTACCCAACTTAATCGCCTTGCAGCACATCCCCCTTTCGCCAGCTGGCGTAATAGCGAAG

 2460  AGGCCCGCACCGATCGCCCTTCCCAACAGTTGCGCAGCCTGAATGGCGAATGGCGAGCTT

 2520  GGCTGTTTTGGCGGATGAGAGAAGATTTTCAGCCTGATACAGATTAAATCAGAACGCAGA

 2580  AGCGGTCTGATAAAACAGAATTTGCCTGGCGGCAGTAGCGCGGTGGTCCCACCTGACCCC

 2640  ATGCCGAACTCAGAAGTGAAACGCCGTAGCGCCGATGGTAGTGTGGGGTCTCCCCATGCG

 2700  AGAGTAGGGAACTGCCAGGCATCAAATAAAACGAAAGGCTCAGTCGAAAGACTGGGCCTT

 2760  TCGTTTTATCTGTTGTTTGTCGGTGAACGCTCTCCTGAGTAGGACAAATCCGCCGGGAGC

 2820  GGATTTGAACGTTGCGAAGCAACGGCCCGGAGGGTGGCGGGCAGGACGCCCGCCATAAAC

 2880  TGCCAGGCATCAAATTAAGCAGAAGGCCATCCTGACGGATGGCCTTTTTGCGTTTCTACA

 2940  AACTCTTTTTGTTTATTTTTCTAAATACATTCAAATATGCATGCGCCTGATGCGGTATTT

 3000  TCTCCTTACGCATATCGACATCCGCCCTCACCGCCAGGAACGCAACCGCAGCCTCATCAC

 3060  GCCGGCGCTTCTTGGCCGCGCGGGATTCAACCCACTCGGCCAGCTCGTCGGTGTAGCTCT

 3120  TTGGCATCGTCTCTCGCCTGTCCCCTCAGTTCAGTAATTTCCTGCATTTGCCTGTTTCCA

 3180  GTCGGTAGATATTCCACAAAACAGCAGGGAAGCAGCGCTTTTCCGCTGCATAACCCTGCT

 3240  TCGGGGTCATTATAGCGATTTTTTCGGTATATCCATCCTTTTTCGCACGATATACAGGAT

 3300  TTTGCCAAAGGGTTCGTGTAGACTTTCCTTGGTGTATCCAACGGCGTCAGCCGGGCAGGA

 3360  TAGGTGAAGTAGGCCCACCCGCGAGCGGGTGTTCCTTCTTCACTGTCCCTTATTCGCACC

 3420  TGGCGGTGCTCAACGGGAATCCTGCTCTGCGAGGCTGGCCGGCTACCGCCGGCGTAACAG

 3480  ATGAGGGCAAGCGGATGGCTGATGAAACCAAGCCAACCAGGAAGGGCAGCCCACCTATCA

 3540  AGGTGTACTGCCTTCCAGACGAACGAAGAGCGATTGAGGAAAAGGCGGCGGCGGCCGGCA

 3600  TGAGCCTGTCGGCCTACCTGCTGGCCGTCGGCCAGGGCTACAAAATCACGGGCGTCGTGG

 3660  ACTATGAGCACGTCCGCGAGCTGGCCCGCATCAATGGCGACCTGGGCCGCCTGGGCGGCC

 3720  TGCTGAAACTCTGGCTCACCGACGACCCGCGCACGGCGCGGTTCGGTGATGCCACGATCC

 3780  TCGCCCTGCTGGCGAAGATCGACTCTAGCTAGAGGATCGATCCTTTTTAACCCATCACAT

 3840  ATACCTGCCGTTCACTATTATTTAGTGAAATGAGATATTATGATATTTTCTGAATTGTGA

 3900  TTAAAAAGGCAACTTTATGCCCATGCAACAGAAACTATAAAAAATACAGAGAATGAAAAG

 3960  AAACAGATAGATTTTTTAGTTCTTTAGGCCCGTAGTCTGCAAATCCTTTTATGATTTTCT

 4020  ATCAAACAAAAGAGGAAAATAGACCAGTTGCAATCCAAACGAGAGTCTAATAGAATGAGG

 4080  TCGAAAAGTAAATCGCGCGGGTTTGTTACTGATAAAGCAGGCAAGACCTAAAATGTGTAA

 4140  AGGGCAAAGTGTATACTTTGGCGTCACCCCTTACATATTTTAGGTCTTTTTTTATTGTGC

 4200  GTAACTAACTTGCCATCTTCAAACAGGAGGGCTGGAAGAAGCAGACCGCTAACACAGTAC

 4260  ATAAAAAAGGAGACATGAACGATGAACATCAAAAAGTTTGCAAAACAAGCAACAGTATTA

    1                       M  N  I  K  K  F  A  K  Q  A  T  V  L

**sacB >>**

 4320  ACCTTTACTACCGCACTGCTGGCAGGAGGCGCAACTCAAGCGTTTGCGAAAGAAACGAAC

    1  T  F  T  T  A  L  L  A  G  G  A  T  Q  A  F  A  K  E  T  N

 4380  CAAAAGCCATATAAGGAAACATACGGCATTTCCCATATTACACGCCATGATATGCTGCAA

    1  Q  K  P  Y  K  E  T  Y  G  I  S  H  I  T  R  H  D  M  L  Q

 4440  ATCCCTGAACAGCAAAAAAATGAAAAATATCAAGTTCCTGAGTTCGATTCGTCCACAATT

    1  I  P  E  Q  Q  K  N  E  K  Y  Q  V  P  E  F  D  S  S  T  I

 4500  AAAAATATCTCTTCTGCAAAAGGCCTGGACGTTTGGGACAGCTGGCCATTACAAAACGCT

    1  K  N  I  S  S  A  K  G  L  D  V  W  D  S  W  P  L  Q  N  A

 4560  GACGGCACTGTCGCAAACTATCACGGCTACCACATCGTCTTTGCATTAGCCGGAGATCCT

    1  D  G  T  V  A  N  Y  H  G  Y  H  I  V  F  A  L  A  G  D  P

 4620  AAAAATGCGGATGACACATCGATTTACATGTTCTATCAAAAAGTCGGCGAAACTTCTATT

    1  K  N  A  D  D  T  S  I  Y  M  F  Y  Q  K  V  G  E  T  S  I

 4680  GACAGCTGGAAAAACGCTGGCCGCGTCTTTAAAGACAGCGACAAATTCGATGCAAATGAT

    1  D  S  W  K  N  A  G  R  V  F  K  D  S  D  K  F  D  A  N  D

 4740  TCTATCCTAAAAGACCAAACACAAGAATGGTCAGGTTCAGCCACATTTACATCTGACGGA

    1  S  I  L  K  D  Q  T  Q  E  W  S  G  S  A  T  F  T  S  D  G

 4800  AAAATCCGTTTATTCTACACTGATTTCTCCGGTAAACATTACGGCAAACAAACACTGACA

    1  K  I  R  L  F  Y  T  D  F  S  G  K  H  Y  G  K  Q  T  L  T

 4860  ACTGCACAAGTTAACGTATCAGCATCAGACAGCTCTTTGAACATCAACGGTGTAGAGGAT

    1  T  A  Q  V  N  V  S  A  S  D  S  S  L  N  I  N  G  V  E  D

 4920  TATAAATCAATCTTTGACGGTGACGGAAAAACGTATCAAAATGTACAGCAGTTCATCGAT

    1  Y  K  S  I  F  D  G  D  G  K  T  Y  Q  N  V  Q  Q  F  I  D

 4980  GAAGGCAACTACAGCTCAGGCGACAACCATACGCTGAGAGATCCTCACTACGTAGAAGAT

    1  E  G  N  Y  S  S  G  D  N  H  T  L  R  D  P  H  Y  V  E  D

 5040  AAAGGCCACAAATACTTAGTATTTGAAGCAAACACTGGAACTGAAGATGGCTACCAAGGC

    1  K  G  H  K  Y  L  V  F  E  A  N  T  G  T  E  D  G  Y  Q  G

 5100  GAAGAATCTTTATTTAACAAAGCATACTATGGCAAAAGCACATCATTCTTCCGTCAAGAA

    1  E  E  S  L  F  N  K  A  Y  Y  G  K  S  T  S  F  F  R  Q  E

 5160  AGTCAAAAACTTCTGCAAAGCGATAAAAAACGCACGGCTGAGTTAGCAAACGGCGCTCTC

    1  S  Q  K  L  L  Q  S  D  K  K  R  T  A  E  L  A  N  G  A  L

 5220  GGTATGATTGAGCTAAACGATGATTACACACTGAAAAAAGTGATGAAACCGCTGATTGCA

    1  G  M  I  E  L  N  D  D  Y  T  L  K  K  V  M  K  P  L  I  A

 5280  TCTAACACAGTAACAGATGAAATTGAACGCGCGAACGTCTTTAAAATGAACGGCAAATGG

    1  S  N  T  V  T  D  E  I  E  R  A  N  V  F  K  M  N  G  K  W

 5340  TATCTGTTCACTGACTCCCGCGGATCAAAAATGACGATTGACGGCATTACGTCTAACGAT

    1  Y  L  F  T  D  S  R  G  S  K  M  T  I  D  G  I  T  S  N  D

 5400  ATTTACATGCTTGGTTATGTTTCTAATTCTTTAACTGGCCCATACAAGCCGCTGAACAAA

    1  I  Y  M  L  G  Y  V  S  N  S  L  T  G  P  Y  K  P  L  N  K

 5460  ACTGGCCTTGTGTTAAAAATGGATCTTGATCCTAACGATGTAACCTTTACTTACTCACAC

    1  T  G  L  V  L  K  M  D  L  D  P  N  D  V  T  F  T  Y  S  H

 5520  TTCGCTGTACCTCAAGCGAAAGGAAACAATGTCGTGATTACAAGCTATATGACAAACAGA

    1  F  A  V  P  Q  A  K  G  N  N  V  V  I  T  S  Y  M  T  N  R

 5580  GGATTCTACGCAGACAAACAATCAACGTTTGCGCCTAGCTTCCTGCTGAACATCAAAGGC

    1  G  F  Y  A  D  K  Q  S  T  F  A  P  S  F  L  L  N  I  K  G

 5640  AAGAAAACATCTGTTGTCAAAGACAGCATCCTTGAACAAGGACAATTAACAGTTAACAAA

    1  K  K  T  S  V  V  K  D  S  I  L  E  Q  G  Q  L  T  V  N  K

 5700  TAAAAACGCAAAAGAAAATGCCGATTATGGTGCACTCTCAGTACAATCTGCTCTGATGCC

 5760  GCATAGTTAAGCCAGCCCCGACACCCGCCAACACCCGCTGACGCGCCCTGACGGGCTTGT

 5820  CTGCTCCCGGCATCCGCTTACAGACAAGCTGTGACCGTCTCCGGGAGCTGCATGTGTCAG

 5880  AGGTTTTCACCGTCATCACCGAAACGCGCGA

**pDOC-K**

0  GACGAAAGGGCCTCGTGATACGCCTATTTTTATAGGTTAATGTCATGATAATAATGGTTT

   60  CTTAGACGTCAGGTGGCACTTTTCGGGGAAATGTGCGCGGAACCCCTATTTGTTTATTTT

  120  TCTAAATACATTCAAATATGTATCCGCTCATGAGACAATAACCCTGATAAATGCTTCAAT

  180  AATATTGAAAAAGGAAGAGTATGAGTATTCAACATTTCCGTGTCGCCCTTATTCCCTTTT

    3                      M  S  I  Q  H  F  R  V  A  L  I  P  F  F

**bla >>**

  240  TTGCGGCATTTTGCCTTCCTGTTTTTGCTCACCCAGAAACGCTGGTGAAAGTAAAAGATG

    3    A  A  F  C  L  P  V  F  A  H  P  E  T  L  V  K  V  K  D  A

  300  CTGAAGATCAGTTGGGTGCACGAGTGGGTTACATCGAACTGGATCTCAACAGCGGTAAGA

    3    E  D  Q  L  G  A  R  V  G  Y  I  E  L  D  L  N  S  G  K  I

  360  TCCTTGAGAGTTTTCGCCCCGAAGAACGTTTTCCAATGATGAGCACTTTTAAAGTTCTGC

    3    L  E  S  F  R  P  E  E  R  F  P  M  M  S  T  F  K  V  L  L

  420  TATGTGGCGCGGTATTATCCCGTATTGACGCCGGGCAAGAGCAACTCGGTCGCCGCATAC

    3    C  G  A  V  L  S  R  I  D  A  G  Q  E  Q  L  G  R  R  I  H

  480  ACTATTCTCAGAATGACTTGGTTGAGTACTCACCAGTCACAGAAAAGCATCTTACGGATG

    3    Y  S  Q  N  D  L  V  E  Y  S  P  V  T  E  K  H  L  T  D  G

  540  GCATGACAGTAAGAGAATTATGCAGTGCTGCCATAACCATGAGTGATAACACTGCGGCCA

    3    M  T  V  R  E  L  C  S  A  A  I  T  M  S  D  N  T  A  A  N

  600  ACTTACTTCTGACAACGATCGGAGGACCGAAGGAGCTAACCGCTTTTTTGCACAACATGG

    3    L  L  L  T  T  I  G  G  P  K  E  L  T  A  F  L  H  N  M  G

  660  GGGATCATGTAACTCGCCTTGATCGTTGGGAACCGGAGCTGAATGAAGCCATACCAAACG

    3    D  H  V  T  R  L  D  R  W  E  P  E  L  N  E  A  I  P  N  D

  720  ACGAGCGTGACACCACGATGCCTGTAGCAATGGCAACAACGTTGCGCAAACTATTAACTG

    3    E  R  D  T  T  M  P  V  A  M  A  T  T  L  R  K  L  L  T  G

  780  GCGAACTACTTACTCTAGCTTCCCGGCAACAATTAATAGACTGGATGGAGGCGGATAAAG

    3    E  L  L  T  L  A  S  R  Q  Q  L  I  D  W  M  E  A  D  K  V

  840  TTGCAGGACCACTTCTGCGCTCGGCCCTTCCGGCTGGCTGGTTTATTGCTGATAAATCTG

    3    A  G  P  L  L  R  S  A  L  P  A  G  W  F  I  A  D  K  S  G

  900  GAGCCGGTGAGCGTGGGTCTCGCGGTATCATTGCAGCACTGGGGCCAGATGGTAAGCCCT

    3    A  G  E  R  G  S  R  G  I  I  A  A  L  G  P  D  G  K  P  S

  960  CCCGTATCGTAGTTATCTACACGACGGGGAGTCAGGCAACTATGGATGAACGAAATAGAC

    3    R  I  V  V  I  Y  T  T  G  S  Q  A  T  M  D  E  R  N  R  Q

 1020  AGATCGCTGAGATAGGTGCCTCACTGATTAAGCATTGGTAACTGTCAGACCAAGTTTACT

    3    I  A  E  I  G  A  S  L  I  K  H  W

 1080  CATATATACTTTAGATTGATTTAAAACTTCATTTTTAATTTAAAAGGATCTAGGTGAAGA

 1140  TCCTTTTTGATAATCTCATGACCAAAATCCCTTAACGTGAGTTTTCGTTCCACTGAGCGT

 1200  CAGACCCCGTAGAAAAGATCAAAGGATCTTCTTGAGATCCTTTTTTTCTGCGCGTAATCT

 1260  GCTGCTTGCAAACAAAAAAACCACCGCTACCAGCGGTGGTTTGTTTGCCGGATCAAGAGC

 1320  TACCAACTCTTTTTCCGAAGGTAACTGGCTTCAGCAGAGCGCAGATACCAAATACTGTCC

 1380  TTCTAGTGTAGCCGTAGTTAGGCCACCACTTCAAGAACTCTGTAGCACCGCCTACATACC

 1440  TCGCTCTGCTAATCCTGTTACCAGTGGCTGCTGCCAGTGGCGATAAGTCGTGTCTTACCG

 1500  GGTTGGACTCAAGACGATAGTTACCGGATAAGGCGCAGCGGTCGGGCTGAACGGGGGGTT

 1560  CGTGCACACAGCCCAGCTTGGAGCGAACGACCTACACCGAACTGAGATACCTACAGCGTG

 1620  AGCTATGAGAAAGCGCCACGCTTCCCGAAGGGAGAAAGGCGGACAGGTATCCGGTAAGCG

 1680  GCAGGGTCGGAACAGGAGAGCGCACGAGGGAGCTTCCAGGGGGAAACGCCTGGTATCTTT

 1740  ATAGTCCTGTCGGGTTTCGCCACCTCTGACTTGAGCGTCGATTTTTGTGATGCTCGTCAG

 1800  GGGGGCGGAGCCTATGGAAAAACGCCAGCAACGCGGCCTTTTTACGGTTCCTGGCCTTTT

 1860  GCTGGCCTTTTGCTCACATGTTCTTTCCTGCGTTATCCCCTGATTCTGTGGATAACCGTA

 1920  TTACCGCCTTTGAGTGAGCTGATACCGCTCGCCGCAGCCGAACGACCGAGCGCAGCGAGT

 1980  CAGTGAGCGAGGAAGCGGAAGAGCGCCCAATACGCAAACCGCCTCTCCCCGCGCGTTGGC

 2040  CGATTCATTAATGCAGCTGGCACGACAGGTTTCCCGACTGGAAAGCGGGCAGTGAGCGCA

 2100  ACGCAATTAATGTGAGTTAGCTCACTCATTAGGCACCCCAGGCTTTACACTTTATGCTTC

 2160  CGGCTCGTATGTTGTGTGGAATTGTGAGCGGATAACAATTTCACACAGGAAACAGCTATG

*HindIII*

SceI **EcoRI** BamHI

 2220  ACCATGATTACGCCAAGCTCTAGGGATAACAGGGTAATCGAT**GAATTC***AAGCTT*GGAT**CC**

**XmaI** *KpnI*

**SmaI** BlnI   **AgeI**  MunI                        flp1

 2280  **CGGG**TA*CCTAGG***ACCGGT**CAATTGGCTGGAGCTGCTTCGAAGTTCCTATACTTTCTAGAG

 2340  AATAGGAACTTCGGAATAGGAACTTCAAGATCCCCCACGCTGCCGCAAGCACTCAGGGCG

**AflII**

 2400  CAAGGGCTGCTAAAGGAAGCGGAACACGTAGAA**CTTAAG**GGAATTGCCAGCTGGGGCGCC

 2460  CTCTGGTAAGGTTGGGAAGCCCTGCAAAGTAAACTGGATGGCTTTCTTGCCGCCAAGGAT

                           BglII

**BclI**

 2520  CTGATGGCGCAGGGGATCAAGATC**TGATCA**AGAGACAGGATGAGGATCGTTTCGCATGAT

    2                                                         **M**  I

**Kan >**

 2580  TGAACAAGATGGATTGCACGCAGGTTCTCCGGCCGCTTGGGTGGAGAGGCTATTCGGCTA

    2   E  Q  D  G  L  H  A  G  S  P  A  A  W  V  E  R  L  F  G  Y

 2640  TGACTGGGCACAACAGACAATCGGCTGCTCTGATGCCGCCGTGTTCCGGCTGTCAGCGCA

    2   D  W  A  Q  Q  T  I  G  C  S  D  A  A  V  F  R  L  S  A  Q

 2700  GGGGCGCCCGGTTCTTTTTGTCAAGACCGACCTGTCCGGTGCCCTGAATGAACTGCAGGA

    2   G  R  P  V  L  F  V  K  T  D  L  S  G  A  L  N  E  L  Q  D

 2760  CGAGGCAGCGCGGCTATCGTGGCTGGCCACGACGGGCGTTCCTTGCGCAGCTGTGCTCGA

    2   E  A  A  R  L  S  W  L  A  T  T  G  V  P  C  A  A  V  L  D

 2820  CGTTGTCACTGAAGCGGGAAGGGACTGGCTGCTATTGGGCGAAGTGCCGGGGCAGGATCT

    2   V  V  T  E  A  G  R  D  W  L  L  L  G  E  V  P  G  Q  D  L

 2880  CCTGTCATCTCACCTTGCTCCTGCCGAGAAAGTATCCATCATGGCTGATGCAATGCGGCG

    2   L  S  S  H  L  A  P  A  E  K  V  S  I  M  A  D  A  M  R  R

 2940  GCTGCATACGCTTGATCCGGCTACCTGCCCATTCGACCACCAAGCGAAACATCGCATCGA

    2   L  H  T  L  D  P  A  T  C  P  F  D  H  Q  A  K  H  R  I  E

 3000  GCGAGCACGTACTCGGATGGAAGCCGGTCTTGTCGATCAGGATGATCTGGACGAAGAGCA

    2   R  A  R  T  R  M  E  A  G  L  V  D  Q  D  D  L  D  E  E  H

 3060  TCAGGGGCTCGCGCCAGCCGAACTGTTCGCCAGGCTCAAGGCGCGCATGCCCGACGGCGA

    2   Q  G  L  A  P  A  E  L  F  A  R  L  K  A  R  M  P  D  G  E

 3120  GGATCTCGTCGTGACCCATGGCGATGCCTGCTTGCCGAATATCATGGTGGAAAATGGCCG

    2   D  L  V  V  T  H  G  D  A  C  L  P  N  I  M  V  E  N  G  R

 3180  CTTTTCTGGATTCATCGACTGTGGCCGGCTGGGTGTGGCGGACCGCTATCAGGACATAGC

    2   F  S  G  F  I  D  C  G  R  L  G  V  A  D  R  Y  Q  D  I  A

 3240  GTTGGCTACCCGTGATATTGCTGAAGAGCTTGGCGGCGAATGGGCTGACCGCTTCCTCGT

    2   L  A  T  R  D  I  A  E  E  L  G  G  E  W  A  D  R  F  L  V

 3300  GCTCTACGGTATCGCCGCTCCCGATTCGCAGCGCATCGCCTTCTATCGCCTTCTTGACGA

    2   L  Y  G  I  A  A  P  D  S  Q  R  I  A  F  Y  R  L  L  D  E

 3360  GTTCTTCTGAGCGGGACTCTGGGGTTCGAAATGACCGACCAAGCGACGCCCAACCTGCCA

    2   F  F

 3420  TCACGAGATTTCGATTCCACCGCCGCCTTCTATGAAAGGTTGGGCTTCGGAATCGTTTTC

 3480  CGGGACGCCGGCTGGATGATCCTCCAGCGCGGGGATCTCATGCTGGAGTTCTTCGCCCAC

flp

 3540  CCCAGCTTCAAAAGCGCTCTGAAGTTCCTATACTTTCTAGAGAATAGGAACTTCGGAATA

**SacI**   **NheI**    *SpeI*

                               XhoI    NdeI       SalI         SceI

 3600  GGAACTAAGGAGGATATTCATATCTC**GAGCT*C****ATAT****G*CTAGC**GTCG*ACTAGT*AGGGATAA

 3660  CAGGGTAATGAGCTTGGCACTGGCCGTCGTTTTACAACGTCGTGACTGGGAAAACCCTGG

 3720  CGTTACCCAACTTAATCGCCTTGCAGCACATCCCCCTTTCGCCAGCTGGCGTAATAGCGA

 3780  AGAGGCCCGCACCGATCGCCCTTCCCAACAGTTGCGCAGCCTGAATGGCGAATGGCGAGC

 3840  TTGGCTGTTTTGGCGGATGAGAGAAGATTTTCAGCCTGATACAGATTAAATCAGAACGCA

 3900  GAAGCGGTCTGATAAAACAGAATTTGCCTGGCGGCAGTAGCGCGGTGGTCCCACCTGACC

 3960  CCATGCCGAACTCAGAAGTGAAACGCCGTAGCGCCGATGGTAGTGTGGGGTCTCCCCATG

 4020  CGAGAGTAGGGAACTGCCAGGCATCAAATAAAACGAAAGGCTCAGTCGAAAGACTGGGCC

 4080  TTTCGTTTTATCTGTTGTTTGTCGGTGAACGCTCTCCTGAGTAGGACAAATCCGCCGGGA

 4140  GCGGATTTGAACGTTGCGAAGCAACGGCCCGGAGGGTGGCGGGCAGGACGCCCGCCATAA

 4200  ACTGCCAGGCATCAAATTAAGCAGAAGGCCATCCTGACGGATGGCCTTTTTGCGTTTCTA

 4260  CAAACTCTTTTTGTTTATTTTTCTAAATACATTCAAATATGCATGCGCCTGATGCGGTAT

 4320  TTTCTCCTTACGCATATCGACATCCGCCCTCACCGCCAGGAACGCAACCGCAGCCTCATC

 4380  ACGCCGGCGCTTCTTGGCCGCGCGGGATTCAACCCACTCGGCCAGCTCGTCGGTGTAGCT

 4440  CTTTGGCATCGTCTCTCGCCTGTCCCCTCAGTTCAGTAATTTCCTGCATTTGCCTGTTTC

 4500  CAGTCGGTAGATATTCCACAAAACAGCAGGGAAGCAGCGCTTTTCCGCTGCATAACCCTG

 4560  CTTCGGGGTCATTATAGCGATTTTTTCGGTATATCCATCCTTTTTCGCACGATATACAGG

 4620  ATTTTGCCAAAGGGTTCGTGTAGACTTTCCTTGGTGTATCCAACGGCGTCAGCCGGGCAG

 4680  GATAGGTGAAGTAGGCCCACCCGCGAGCGGGTGTTCCTTCTTCACTGTCCCTTATTCGCA

 4740  CCTGGCGGTGCTCAACGGGAATCCTGCTCTGCGAGGCTGGCCGGCTACCGCCGGCGTAAC

 4800  AGATGAGGGCAAGCGGATGGCTGATGAAACCAAGCCAACCAGGAAGGGCAGCCCACCTAT

 4860  CAAGGTGTACTGCCTTCCAGACGAACGAAGAGCGATTGAGGAAAAGGCGGCGGCGGCCGG

 4920  CATGAGCCTGTCGGCCTACCTGCTGGCCGTCGGCCAGGGCTACAAAATCACGGGCGTCGT

 4980  GGACTATGAGCACGTCCGCGAGCTGGCCCGCATCAATGGCGACCTGGGCCGCCTGGGCGG

 5040  CCTGCTGAAACTCTGGCTCACCGACGACCCGCGCACGGCGCGGTTCGGTGATGCCACGAT

 5100  CCTCGCCCTGCTGGCGAAGATCGACTCTAGCTAGAGGATCGATCCTTTTTAACCCATCAC

 5160  ATATACCTGCCGTTCACTATTATTTAGTGAAATGAGATATTATGATATTTTCTGAATTGT

 5220  GATTAAAAAGGCAACTTTATGCCCATGCAACAGAAACTATAAAAAATACAGAGAATGAAA

 5280  AGAAACAGATAGATTTTTTAGTTCTTTAGGCCCGTAGTCTGCAAATCCTTTTATGATTTT

 5340  CTATCAAACAAAAGAGGAAAATAGACCAGTTGCAATCCAAACGAGAGTCTAATAGAATGA

 5400  GGTCGAAAAGTAAATCGCGCGGGTTTGTTACTGATAAAGCAGGCAAGACCTAAAATGTGT

 5460  AAAGGGCAAAGTGTATACTTTGGCGTCACCCCTTACATATTTTAGGTCTTTTTTTATTGT

 5520  GCGTAACTAACTTGCCATCTTCAAACAGGAGGGCTGGAAGAAGCAGACCGCTAACACAGT

 5580  ACATAAAAAAGGAGACATGAACGATGAACATCAAAAAGTTTGCAAAACAAGCAACAGTAT

    3                         M  N  I  K  K  F  A  K  Q  A  T  V  L

**sacB >>**

 5640  TAACCTTTACTACCGCACTGCTGGCAGGAGGCGCAACTCAAGCGTTTGCGAAAGAAACGA

    3    T  F  T  T  A  L  L  A  G  G  A  T  Q  A  F  A  K  E  T  N

 5700  ACCAAAAGCCATATAAGGAAACATACGGCATTTCCCATATTACACGCCATGATATGCTGC

    3    Q  K  P  Y  K  E  T  Y  G  I  S  H  I  T  R  H  D  M  L  Q

 5760  AAATCCCTGAACAGCAAAAAAATGAAAAATATCAAGTTCCTGAGTTCGATTCGTCCACAA

    3    I  P  E  Q  Q  K  N  E  K  Y  Q  V  P  E  F  D  S  S  T  I

 5820  TTAAAAATATCTCTTCTGCAAAAGGCCTGGACGTTTGGGACAGCTGGCCATTACAAAACG

    3    K  N  I  S  S  A  K  G  L  D  V  W  D  S  W  P  L  Q  N  A

 5880  CTGACGGCACTGTCGCAAACTATCACGGCTACCACATCGTCTTTGCATTAGCCGGAGATC

    3    D  G  T  V  A  N  Y  H  G  Y  H  I  V  F  A  L  A  G  D  P

 5940  CTAAAAATGCGGATGACACATCGATTTACATGTTCTATCAAAAAGTCGGCGAAACTTCTA

    3    K  N  A  D  D  T  S  I  Y  M  F  Y  Q  K  V  G  E  T  S  I

 6000  TTGACAGCTGGAAAAACGCTGGCCGCGTCTTTAAAGACAGCGACAAATTCGATGCAAATG

    3    D  S  W  K  N  A  G  R  V  F  K  D  S  D  K  F  D  A  N  D

 6060  ATTCTATCCTAAAAGACCAAACACAAGAATGGTCAGGTTCAGCCACATTTACATCTGACG

    3    S  I  L  K  D  Q  T  Q  E  W  S  G  S  A  T  F  T  S  D  G

 6120  GAAAAATCCGTTTATTCTACACTGATTTCTCCGGTAAACATTACGGCAAACAAACACTGA

    3    K  I  R  L  F  Y  T  D  F  S  G  K  H  Y  G  K  Q  T  L  T

 6180  CAACTGCACAAGTTAACGTATCAGCATCAGACAGCTCTTTGAACATCAACGGTGTAGAGG

    3    T  A  Q  V  N  V  S  A  S  D  S  S  L  N  I  N  G  V  E  D

 6240  ATTATAAATCAATCTTTGACGGTGACGGAAAAACGTATCAAAATGTACAGCAGTTCATCG

    3    Y  K  S  I  F  D  G  D  G  K  T  Y  Q  N  V  Q  Q  F  I  D

 6300  ATGAAGGCAACTACAGCTCAGGCGACAACCATACGCTGAGAGATCCTCACTACGTAGAAG

    3    E  G  N  Y  S  S  G  D  N  H  T  L  R  D  P  H  Y  V  E  D

 6360  ATAAAGGCCACAAATACTTAGTATTTGAAGCAAACACTGGAACTGAAGATGGCTACCAAG

    3    K  G  H  K  Y  L  V  F  E  A  N  T  G  T  E  D  G  Y  Q  G

 6420  GCGAAGAATCTTTATTTAACAAAGCATACTATGGCAAAAGCACATCATTCTTCCGTCAAG

    3    E  E  S  L  F  N  K  A  Y  Y  G  K  S  T  S  F  F  R  Q  E

 6480  AAAGTCAAAAACTTCTGCAAAGCGATAAAAAACGCACGGCTGAGTTAGCAAACGGCGCTC

    3    S  Q  K  L  L  Q  S  D  K  K  R  T  A  E  L  A  N  G  A  L

 6540  TCGGTATGATTGAGCTAAACGATGATTACACACTGAAAAAAGTGATGAAACCGCTGATTG

    3    G  M  I  E  L  N  D  D  Y  T  L  K  K  V  M  K  P  L  I  A

 6600  CATCTAACACAGTAACAGATGAAATTGAACGCGCGAACGTCTTTAAAATGAACGGCAAAT

    3    S  N  T  V  T  D  E  I  E  R  A  N  V  F  K  M  N  G  K  W

 6660  GGTATCTGTTCACTGACTCCCGCGGATCAAAAATGACGATTGACGGCATTACGTCTAACG

    3    Y  L  F  T  D  S  R  G  S  K  M  T  I  D  G  I  T  S  N  D

 6720  ATATTTACATGCTTGGTTATGTTTCTAATTCTTTAACTGGCCCATACAAGCCGCTGAACA

    3    I  Y  M  L  G  Y  V  S  N  S  L  T  G  P  Y  K  P  L  N  K

 6780  AAACTGGCCTTGTGTTAAAAATGGATCTTGATCCTAACGATGTAACCTTTACTTACTCAC

    3    T  G  L  V  L  K  M  D  L  D  P  N  D  V  T  F  T  Y  S  H

 6840  ACTTCGCTGTACCTCAAGCGAAAGGAAACAATGTCGTGATTACAAGCTATATGACAAACA

    3    F  A  V  P  Q  A  K  G  N  N  V  V  I  T  S  Y  M  T  N  R

 6900  GAGGATTCTACGCAGACAAACAATCAACGTTTGCGCCTAGCTTCCTGCTGAACATCAAAG

    3    G  F  Y  A  D  K  Q  S  T  F  A  P  S  F  L  L  N  I  K  G

 6960  GCAAGAAAACATCTGTTGTCAAAGACAGCATCCTTGAACAAGGACAATTAACAGTTAACA

    3    K  K  T  S  V  V  K  D  S  I  L  E  Q  G  Q  L  T  V  N  K

 7020  AATAAAAACGCAAAAGAAAATGCCGATTATGGTGCACTCTCAGTACAATCTGCTCTGATG

 7080  CCGCATAGTTAAGCCAGCCCCGACACCCGCCAACACCCGCTGACGCGCCCTGACGGGCTT

 7140  GTCTGCTCCCGGCATCCGCTTACAGACAAGCTGTGACCGTCTCCGGGAGCTGCATGTGTC

 7200  AGAGGTTTTCACCGTCATCACCGAAACGCGCGA

**pDOC-H**

0  GACGAAAGGGCCTCGTGATACGCCTATTTTTATAGGTTAATGTCATGATAATAATGGTTT

   60  CTTAGACGTCAGGTGGCACTTTTCGGGGAAATGTGCGCGGAACCCCTATTTGTTTATTTT

  120  TCTAAATACATTCAAATATGTATCCGCTCATGAGACAATAACCCTGATAAATGCTTCAAT

  180  AATATTGAAAAAGGAAGAGTATGAGTATTCAACATTTCCGTGTCGCCCTTATTCCCTTTT

    3                      M  S  I  Q  H  F  R  V  A  L  I  P  F  F

**bla >>**

  240  TTGCGGCATTTTGCCTTCCTGTTTTTGCTCACCCAGAAACGCTGGTGAAAGTAAAAGATG

    3    A  A  F  C  L  P  V  F  A  H  P  E  T  L  V  K  V  K  D  A

  300  CTGAAGATCAGTTGGGTGCACGAGTGGGTTACATCGAACTGGATCTCAACAGCGGTAAGA

    3    E  D  Q  L  G  A  R  V  G  Y  I  E  L  D  L  N  S  G  K  I

  360  TCCTTGAGAGTTTTCGCCCCGAAGAACGTTTTCCAATGATGAGCACTTTTAAAGTTCTGC

    3    L  E  S  F  R  P  E  E  R  F  P  M  M  S  T  F  K  V  L  L

  420  TATGTGGCGCGGTATTATCCCGTATTGACGCCGGGCAAGAGCAACTCGGTCGCCGCATAC

    3    C  G  A  V  L  S  R  I  D  A  G  Q  E  Q  L  G  R  R  I  H

  480  ACTATTCTCAGAATGACTTGGTTGAGTACTCACCAGTCACAGAAAAGCATCTTACGGATG

    3    Y  S  Q  N  D  L  V  E  Y  S  P  V  T  E  K  H  L  T  D  G

  540  GCATGACAGTAAGAGAATTATGCAGTGCTGCCATAACCATGAGTGATAACACTGCGGCCA

    3    M  T  V  R  E  L  C  S  A  A  I  T  M  S  D  N  T  A  A  N

  600  ACTTACTTCTGACAACGATCGGAGGACCGAAGGAGCTAACCGCTTTTTTGCACAACATGG

    3    L  L  L  T  T  I  G  G  P  K  E  L  T  A  F  L  H  N  M  G

  660  GGGATCATGTAACTCGCCTTGATCGTTGGGAACCGGAGCTGAATGAAGCCATACCAAACG

    3    D  H  V  T  R  L  D  R  W  E  P  E  L  N  E  A  I  P  N  D

  720  ACGAGCGTGACACCACGATGCCTGTAGCAATGGCAACAACGTTGCGCAAACTATTAACTG

    3    E  R  D  T  T  M  P  V  A  M  A  T  T  L  R  K  L  L  T  G

  780  GCGAACTACTTACTCTAGCTTCCCGGCAACAATTAATAGACTGGATGGAGGCGGATAAAG

    3    E  L  L  T  L  A  S  R  Q  Q  L  I  D  W  M  E  A  D  K  V

  840  TTGCAGGACCACTTCTGCGCTCGGCCCTTCCGGCTGGCTGGTTTATTGCTGATAAATCTG

    3    A  G  P  L  L  R  S  A  L  P  A  G  W  F  I  A  D  K  S  G

  900  GAGCCGGTGAGCGTGGGTCTCGCGGTATCATTGCAGCACTGGGGCCAGATGGTAAGCCCT

    3    A  G  E  R  G  S  R  G  I  I  A  A  L  G  P  D  G  K  P  S

  960  CCCGTATCGTAGTTATCTACACGACGGGGAGTCAGGCAACTATGGATGAACGAAATAGAC

    3    R  I  V  V  I  Y  T  T  G  S  Q  A  T  M  D  E  R  N  R  Q

 1020  AGATCGCTGAGATAGGTGCCTCACTGATTAAGCATTGGTAACTGTCAGACCAAGTTTACT

    3    I  A  E  I  G  A  S  L  I  K  H  W

 1080  CATATATACTTTAGATTGATTTAAAACTTCATTTTTAATTTAAAAGGATCTAGGTGAAGA

 1140  TCCTTTTTGATAATCTCATGACCAAAATCCCTTAACGTGAGTTTTCGTTCCACTGAGCGT

 1200  CAGACCCCGTAGAAAAGATCAAAGGATCTTCTTGAGATCCTTTTTTTCTGCGCGTAATCT

 1260  GCTGCTTGCAAACAAAAAAACCACCGCTACCAGCGGTGGTTTGTTTGCCGGATCAAGAGC

 1320  TACCAACTCTTTTTCCGAAGGTAACTGGCTTCAGCAGAGCGCAGATACCAAATACTGTCC

 1380  TTCTAGTGTAGCCGTAGTTAGGCCACCACTTCAAGAACTCTGTAGCACCGCCTACATACC

 1440  TCGCTCTGCTAATCCTGTTACCAGTGGCTGCTGCCAGTGGCGATAAGTCGTGTCTTACCG

 1500  GGTTGGACTCAAGACGATAGTTACCGGATAAGGCGCAGCGGTCGGGCTGAACGGGGGGTT

 1560  CGTGCACACAGCCCAGCTTGGAGCGAACGACCTACACCGAACTGAGATACCTACAGCGTG

 1620  AGCTATGAGAAAGCGCCACGCTTCCCGAAGGGAGAAAGGCGGACAGGTATCCGGTAAGCG

 1680  GCAGGGTCGGAACAGGAGAGCGCACGAGGGAGCTTCCAGGGGGAAACGCCTGGTATCTTT

 1740  ATAGTCCTGTCGGGTTTCGCCACCTCTGACTTGAGCGTCGATTTTTGTGATGCTCGTCAG

 1800  GGGGGCGGAGCCTATGGAAAAACGCCAGCAACGCGGCCTTTTTACGGTTCCTGGCCTTTT

 1860  GCTGGCCTTTTGCTCACATGTTCTTTCCTGCGTTATCCCCTGATTCTGTGGATAACCGTA

 1920  TTACCGCCTTTGAGTGAGCTGATACCGCTCGCCGCAGCCGAACGACCGAGCGCAGCGAGT

 1980  CAGTGAGCGAGGAAGCGGAAGAGCGCCCAATACGCAAACCGCCTCTCCCCGCGCGTTGGC

 2040  CGATTCATTAATGCAGCTGGCACGACAGGTTTCCCGACTGGAAAGCGGGCAGTGAGCGCA

 2100  ACGCAATTAATGTGAGTTAGCTCACTCATTAGGCACCCCAGGCTTTACACTTTATGCTTC

 2160  CGGCTCGTATGTTGTGTGGAATTGTGAGCGGATAACAATTTCACACAGGAAACAGCTATG

*HindIII*

SceI **EcoRI** BamHI

 2220  ACCATGATTACGCCAAGCTCTAGGGATAACAGGGTAATCGAT**GAATTC***AAGCTT*GGAT**CC**

**XmaI** KpnI

**SmaI**  **6xHis** **STOP** AgeI  **MunI**

 2280  **CGGG**TACC**CACCATCACCATCACCATTAA**ACCGGT**CAATTG**GCTGGAGCTGCTTCGAAGT

Flp1

 2340  TCCTATACTTTCTAGAGAATAGGAACTTCGGAATAGGAACTTCAAGATCCCCCACGCTGC

 2400  CGCAAGCACTCAGGGCGCAAGGGCTGCTAAAGGAAGCGGAACACGTAGAACTTAAGGGAA

 2460  TTGCCAGCTGGGGCGCCCTCTGGTAAGGTTGGGAAGCCCTGCAAAGTAAACTGGATGGCT

 2520  TTCTTGCCGCCAAGGATCTGATGGCGCAGGGGATCAAGATCTGATCAAGAGACAGGATGA

 2580  GGATCGTTTCGCATGATTGAACAAGATGGATTGCACGCAGGTTCTCCGGCCGCTTGGGTG

    1              **M**  I  E  Q  D  G  L  H  A  G  S  P  A  A  W  V

**Kan >**

 2640  GAGAGGCTATTCGGCTATGACTGGGCACAACAGACAATCGGCTGCTCTGATGCCGCCGTG

    1  E  R  L  F  G  Y  D  W  A  Q  Q  T  I  G  C  S  D  A  A  V

 2700  TTCCGGCTGTCAGCGCAGGGGCGCCCGGTTCTTTTTGTCAAGACCGACCTGTCCGGTGCC

    1  F  R  L  S  A  Q  G  R  P  V  L  F  V  K  T  D  L  S  G  A

 2760  CTGAATGAACTGCAGGACGAGGCAGCGCGGCTATCGTGGCTGGCCACGACGGGCGTTCCT

    1  L  N  E  L  Q  D  E  A  A  R  L  S  W  L  A  T  T  G  V  P

 2820  TGCGCAGCTGTGCTCGACGTTGTCACTGAAGCGGGAAGGGACTGGCTGCTATTGGGCGAA

    1  C  A  A  V  L  D  V  V  T  E  A  G  R  D  W  L  L  L  G  E

 2880  GTGCCGGGGCAGGATCTCCTGTCATCTCACCTTGCTCCTGCCGAGAAAGTATCCATCATG

    1  V  P  G  Q  D  L  L  S  S  H  L  A  P  A  E  K  V  S  I  M

 2940  GCTGATGCAATGCGGCGGCTGCATACGCTTGATCCGGCTACCTGCCCATTCGACCACCAA

    1  A  D  A  M  R  R  L  H  T  L  D  P  A  T  C  P  F  D  H  Q

 3000  GCGAAACATCGCATCGAGCGAGCACGTACTCGGATGGAAGCCGGTCTTGTCGATCAGGAT

    1  A  K  H  R  I  E  R  A  R  T  R  M  E  A  G  L  V  D  Q  D

 3060  GATCTGGACGAAGAGCATCAGGGGCTCGCGCCAGCCGAACTGTTCGCCAGGCTCAAGGCG

    1  D  L  D  E  E  H  Q  G  L  A  P  A  E  L  F  A  R  L  K  A

 3120  CGCATGCCCGACGGCGAGGATCTCGTCGTGACCCATGGCGATGCCTGCTTGCCGAATATC

    1  R  M  P  D  G  E  D  L  V  V  T  H  G  D  A  C  L  P  N  I

 3180  ATGGTGGAAAATGGCCGCTTTTCTGGATTCATCGACTGTGGCCGGCTGGGTGTGGCGGAC

    1  M  V  E  N  G  R  F  S  G  F  I  D  C  G  R  L  G  V  A  D

 3240  CGCTATCAGGACATAGCGTTGGCTACCCGTGATATTGCTGAAGAGCTTGGCGGCGAATGG

    1  R  Y  Q  D  I  A  L  A  T  R  D  I  A  E  E  L  G  G  E  W

 3300  GCTGACCGCTTCCTCGTGCTCTACGGTATCGCCGCTCCCGATTCGCAGCGCATCGCCTTC

    1  A  D  R  F  L  V  L  Y  G  I  A  A  P  D  S  Q  R  I  A  F

 3360  TATCGCCTTCTTGACGAGTTCTTCTGAGCGGGACTCTGGGGTTCGAAATGACCGACCAAG

    1  Y  R  L  L  D  E  F  F

 3420  CGACGCCCAACCTGCCATCACGAGATTTCGATTCCACCGCCGCCTTCTATGAAAGGTTGG

 3480  GCTTCGGAATCGTTTTCCGGGACGCCGGCTGGATGATCCTCCAGCGCGGGGATCTCATGC

Flp2

 3540  TGGAGTTCTTCGCCCACCCCAGCTTCAAAAGCGCTCTGAAGTTCCTATACTTTCTAGAGA

**SacI**   **NheI**

                                XhoI    *NdeI*

 3600  ATAGGAACTTCGGAATAGGAACTAAGGAGGATATTCATATCTC**GAGCT*C****ATAT****G*CTAGC**G

*SpeI*

       SalI SceI

 3660  TCG*ACTAGT*AGGGATAACAGGGTAATGAGCTTGGCACTGGCCGTCGTTTTACAACGTCGT

 3720  GACTGGGAAAACCCTGGCGTTACCCAACTTAATCGCCTTGCAGCACATCCCCCTTTCGCC

 3780  AGCTGGCGTAATAGCGAAGAGGCCCGCACCGATCGCCCTTCCCAACAGTTGCGCAGCCTG

 3840  AATGGCGAATGGCGAGCTTGGCTGTTTTGGCGGATGAGAGAAGATTTTCAGCCTGATACA

 3900  GATTAAATCAGAACGCAGAAGCGGTCTGATAAAACAGAATTTGCCTGGCGGCAGTAGCGC

 3960  GGTGGTCCCACCTGACCCCATGCCGAACTCAGAAGTGAAACGCCGTAGCGCCGATGGTAG

 4020  TGTGGGGTCTCCCCATGCGAGAGTAGGGAACTGCCAGGCATCAAATAAAACGAAAGGCTC

 4080  AGTCGAAAGACTGGGCCTTTCGTTTTATCTGTTGTTTGTCGGTGAACGCTCTCCTGAGTA

 4140  GGACAAATCCGCCGGGAGCGGATTTGAACGTTGCGAAGCAACGGCCCGGAGGGTGGCGGG

 4200  CAGGACGCCCGCCATAAACTGCCAGGCATCAAATTAAGCAGAAGGCCATCCTGACGGATG

 4260  GCCTTTTTGCGTTTCTACAAACTCTTTTTGTTTATTTTTCTAAATACATTCAAATATGCA

 4320  TGCGCCTGATGCGGTATTTTCTCCTTACGCATATCGACATCCGCCCTCACCGCCAGGAAC

 4380  GCAACCGCAGCCTCATCACGCCGGCGCTTCTTGGCCGCGCGGGATTCAACCCACTCGGCC

 4440  AGCTCGTCGGTGTAGCTCTTTGGCATCGTCTCTCGCCTGTCCCCTCAGTTCAGTAATTTC

 4500  CTGCATTTGCCTGTTTCCAGTCGGTAGATATTCCACAAAACAGCAGGGAAGCAGCGCTTT

 4560  TCCGCTGCATAACCCTGCTTCGGGGTCATTATAGCGATTTTTTCGGTATATCCATCCTTT

 4620  TTCGCACGATATACAGGATTTTGCCAAAGGGTTCGTGTAGACTTTCCTTGGTGTATCCAA

 4680  CGGCGTCAGCCGGGCAGGATAGGTGAAGTAGGCCCACCCGCGAGCGGGTGTTCCTTCTTC

 4740  ACTGTCCCTTATTCGCACCTGGCGGTGCTCAACGGGAATCCTGCTCTGCGAGGCTGGCCG

 4800  GCTACCGCCGGCGTAACAGATGAGGGCAAGCGGATGGCTGATGAAACCAAGCCAACCAGG

 4860  AAGGGCAGCCCACCTATCAAGGTGTACTGCCTTCCAGACGAACGAAGAGCGATTGAGGAA

 4920  AAGGCGGCGGCGGCCGGCATGAGCCTGTCGGCCTACCTGCTGGCCGTCGGCCAGGGCTAC

 4980  AAAATCACGGGCGTCGTGGACTATGAGCACGTCCGCGAGCTGGCCCGCATCAATGGCGAC

 5040  CTGGGCCGCCTGGGCGGCCTGCTGAAACTCTGGCTCACCGACGACCCGCGCACGGCGCGG

 5100  TTCGGTGATGCCACGATCCTCGCCCTGCTGGCGAAGATCGACTCTAGCTAGAGGATCGAT

 5160  CCTTTTTAACCCATCACATATACCTGCCGTTCACTATTATTTAGTGAAATGAGATATTAT

 5220  GATATTTTCTGAATTGTGATTAAAAAGGCAACTTTATGCCCATGCAACAGAAACTATAAA

 5280  AAATACAGAGAATGAAAAGAAACAGATAGATTTTTTAGTTCTTTAGGCCCGTAGTCTGCA

 5340  AATCCTTTTATGATTTTCTATCAAACAAAAGAGGAAAATAGACCAGTTGCAATCCAAACG

 5400  AGAGTCTAATAGAATGAGGTCGAAAAGTAAATCGCGCGGGTTTGTTACTGATAAAGCAGG

 5460  CAAGACCTAAAATGTGTAAAGGGCAAAGTGTATACTTTGGCGTCACCCCTTACATATTTT

 5520  AGGTCTTTTTTTATTGTGCGTAACTAACTTGCCATCTTCAAACAGGAGGGCTGGAAGAAG

 5580  CAGACCGCTAACACAGTACATAAAAAAGGAGACATGAACGATGAACATCAAAAAGTTTGC

    2                                          M  N  I  K  K  F  A

**sacB >>**

 5640  AAAACAAGCAACAGTATTAACCTTTACTACCGCACTGCTGGCAGGAGGCGCAACTCAAGC

    2   K  Q  A  T  V  L  T  F  T  T  A  L  L  A  G  G  A  T  Q  A

 5700  GTTTGCGAAAGAAACGAACCAAAAGCCATATAAGGAAACATACGGCATTTCCCATATTAC

    2   F  A  K  E  T  N  Q  K  P  Y  K  E  T  Y  G  I  S  H  I  T

 5760  ACGCCATGATATGCTGCAAATCCCTGAACAGCAAAAAAATGAAAAATATCAAGTTCCTGA

    2   R  H  D  M  L  Q  I  P  E  Q  Q  K  N  E  K  Y  Q  V  P  E

 5820  GTTCGATTCGTCCACAATTAAAAATATCTCTTCTGCAAAAGGCCTGGACGTTTGGGACAG

    2   F  D  S  S  T  I  K  N  I  S  S  A  K  G  L  D  V  W  D  S

 5880  CTGGCCATTACAAAACGCTGACGGCACTGTCGCAAACTATCACGGCTACCACATCGTCTT

    2   W  P  L  Q  N  A  D  G  T  V  A  N  Y  H  G  Y  H  I  V  F

 5940  TGCATTAGCCGGAGATCCTAAAAATGCGGATGACACATCGATTTACATGTTCTATCAAAA

    2   A  L  A  G  D  P  K  N  A  D  D  T  S  I  Y  M  F  Y  Q  K

 6000  AGTCGGCGAAACTTCTATTGACAGCTGGAAAAACGCTGGCCGCGTCTTTAAAGACAGCGA

    2   V  G  E  T  S  I  D  S  W  K  N  A  G  R  V  F  K  D  S  D

 6060  CAAATTCGATGCAAATGATTCTATCCTAAAAGACCAAACACAAGAATGGTCAGGTTCAGC

    2   K  F  D  A  N  D  S  I  L  K  D  Q  T  Q  E  W  S  G  S  A

 6120  CACATTTACATCTGACGGAAAAATCCGTTTATTCTACACTGATTTCTCCGGTAAACATTA

    2   T  F  T  S  D  G  K  I  R  L  F  Y  T  D  F  S  G  K  H  Y

 6180  CGGCAAACAAACACTGACAACTGCACAAGTTAACGTATCAGCATCAGACAGCTCTTTGAA

    2   G  K  Q  T  L  T  T  A  Q  V  N  V  S  A  S  D  S  S  L  N

 6240  CATCAACGGTGTAGAGGATTATAAATCAATCTTTGACGGTGACGGAAAAACGTATCAAAA

    2   I  N  G  V  E  D  Y  K  S  I  F  D  G  D  G  K  T  Y  Q  N

 6300  TGTACAGCAGTTCATCGATGAAGGCAACTACAGCTCAGGCGACAACCATACGCTGAGAGA

    2   V  Q  Q  F  I  D  E  G  N  Y  S  S  G  D  N  H  T  L  R  D

 6360  TCCTCACTACGTAGAAGATAAAGGCCACAAATACTTAGTATTTGAAGCAAACACTGGAAC

    2   P  H  Y  V  E  D  K  G  H  K  Y  L  V  F  E  A  N  T  G  T

 6420  TGAAGATGGCTACCAAGGCGAAGAATCTTTATTTAACAAAGCATACTATGGCAAAAGCAC

    2   E  D  G  Y  Q  G  E  E  S  L  F  N  K  A  Y  Y  G  K  S  T

 6480  ATCATTCTTCCGTCAAGAAAGTCAAAAACTTCTGCAAAGCGATAAAAAACGCACGGCTGA

    2   S  F  F  R  Q  E  S  Q  K  L  L  Q  S  D  K  K  R  T  A  E

 6540  GTTAGCAAACGGCGCTCTCGGTATGATTGAGCTAAACGATGATTACACACTGAAAAAAGT

    2   L  A  N  G  A  L  G  M  I  E  L  N  D  D  Y  T  L  K  K  V

 6600  GATGAAACCGCTGATTGCATCTAACACAGTAACAGATGAAATTGAACGCGCGAACGTCTT

    2   M  K  P  L  I  A  S  N  T  V  T  D  E  I  E  R  A  N  V  F

 6660  TAAAATGAACGGCAAATGGTATCTGTTCACTGACTCCCGCGGATCAAAAATGACGATTGA

    2   K  M  N  G  K  W  Y  L  F  T  D  S  R  G  S  K  M  T  I  D

 6720  CGGCATTACGTCTAACGATATTTACATGCTTGGTTATGTTTCTAATTCTTTAACTGGCCC

    2   G  I  T  S  N  D  I  Y  M  L  G  Y  V  S  N  S  L  T  G  P

 6780  ATACAAGCCGCTGAACAAAACTGGCCTTGTGTTAAAAATGGATCTTGATCCTAACGATGT

    2   Y  K  P  L  N  K  T  G  L  V  L  K  M  D  L  D  P  N  D  V

 6840  AACCTTTACTTACTCACACTTCGCTGTACCTCAAGCGAAAGGAAACAATGTCGTGATTAC

    2   T  F  T  Y  S  H  F  A  V  P  Q  A  K  G  N  N  V  V  I  T

 6900  AAGCTATATGACAAACAGAGGATTCTACGCAGACAAACAATCAACGTTTGCGCCTAGCTT

    2   S  Y  M  T  N  R  G  F  Y  A  D  K  Q  S  T  F  A  P  S  F

 6960  CCTGCTGAACATCAAAGGCAAGAAAACATCTGTTGTCAAAGACAGCATCCTTGAACAAGG

    2   L  L  N  I  K  G  K  K  T  S  V  V  K  D  S  I  L  E  Q  G

 7020  ACAATTAACAGTTAACAAATAAAAACGCAAAAGAAAATGCCGATTATGGTGCACTCTCAG

    2   Q  L  T  V  N  K

 7080  TACAATCTGCTCTGATGCCGCATAGTTAAGCCAGCCCCGACACCCGCCAACACCCGCTGA

 7140  CGCGCCCTGACGGGCTTGTCTGCTCCCGGCATCCGCTTACAGACAAGCTGTGACCGTCTC

 7200  CGGGAGCTGCATGTGTCAGAGGTTTTCACCGTCATCACCGAAACGCGCGA

**pDOC-F**

0  GACGAAAGGGCCTCGTGATACGCCTATTTTTATAGGTTAATGTCATGATAATAATGGTTT

   60  CTTAGACGTCAGGTGGCACTTTTCGGGGAAATGTGCGCGGAACCCCTATTTGTTTATTTT

  120  TCTAAATACATTCAAATATGTATCCGCTCATGAGACAATAACCCTGATAAATGCTTCAAT

  180  AATATTGAAAAAGGAAGAGTATGAGTATTCAACATTTCCGTGTCGCCCTTATTCCCTTTT

    3                      M  S  I  Q  H  F  R  V  A  L  I  P  F  F

**bla >>**

  240  TTGCGGCATTTTGCCTTCCTGTTTTTGCTCACCCAGAAACGCTGGTGAAAGTAAAAGATG

    3    A  A  F  C  L  P  V  F  A  H  P  E  T  L  V  K  V  K  D  A

  300  CTGAAGATCAGTTGGGTGCACGAGTGGGTTACATCGAACTGGATCTCAACAGCGGTAAGA

    3    E  D  Q  L  G  A  R  V  G  Y  I  E  L  D  L  N  S  G  K  I

  360  TCCTTGAGAGTTTTCGCCCCGAAGAACGTTTTCCAATGATGAGCACTTTTAAAGTTCTGC

    3    L  E  S  F  R  P  E  E  R  F  P  M  M  S  T  F  K  V  L  L

  420  TATGTGGCGCGGTATTATCCCGTATTGACGCCGGGCAAGAGCAACTCGGTCGCCGCATAC

    3    C  G  A  V  L  S  R  I  D  A  G  Q  E  Q  L  G  R  R  I  H

  480  ACTATTCTCAGAATGACTTGGTTGAGTACTCACCAGTCACAGAAAAGCATCTTACGGATG

    3    Y  S  Q  N  D  L  V  E  Y  S  P  V  T  E  K  H  L  T  D  G

  540  GCATGACAGTAAGAGAATTATGCAGTGCTGCCATAACCATGAGTGATAACACTGCGGCCA

    3    M  T  V  R  E  L  C  S  A  A  I  T  M  S  D  N  T  A  A  N

  600  ACTTACTTCTGACAACGATCGGAGGACCGAAGGAGCTAACCGCTTTTTTGCACAACATGG

    3    L  L  L  T  T  I  G  G  P  K  E  L  T  A  F  L  H  N  M  G

  660  GGGATCATGTAACTCGCCTTGATCGTTGGGAACCGGAGCTGAATGAAGCCATACCAAACG

    3    D  H  V  T  R  L  D  R  W  E  P  E  L  N  E  A  I  P  N  D

  720  ACGAGCGTGACACCACGATGCCTGTAGCAATGGCAACAACGTTGCGCAAACTATTAACTG

    3    E  R  D  T  T  M  P  V  A  M  A  T  T  L  R  K  L  L  T  G

  780  GCGAACTACTTACTCTAGCTTCCCGGCAACAATTAATAGACTGGATGGAGGCGGATAAAG

    3    E  L  L  T  L  A  S  R  Q  Q  L  I  D  W  M  E  A  D  K  V

  840  TTGCAGGACCACTTCTGCGCTCGGCCCTTCCGGCTGGCTGGTTTATTGCTGATAAATCTG

    3    A  G  P  L  L  R  S  A  L  P  A  G  W  F  I  A  D  K  S  G

  900  GAGCCGGTGAGCGTGGGTCTCGCGGTATCATTGCAGCACTGGGGCCAGATGGTAAGCCCT

    3    A  G  E  R  G  S  R  G  I  I  A  A  L  G  P  D  G  K  P  S

  960  CCCGTATCGTAGTTATCTACACGACGGGGAGTCAGGCAACTATGGATGAACGAAATAGAC

    3    R  I  V  V  I  Y  T  T  G  S  Q  A  T  M  D  E  R  N  R  Q

 1020  AGATCGCTGAGATAGGTGCCTCACTGATTAAGCATTGGTAACTGTCAGACCAAGTTTACT

    3    I  A  E  I  G  A  S  L  I  K  H  W

 1080  CATATATACTTTAGATTGATTTAAAACTTCATTTTTAATTTAAAAGGATCTAGGTGAAGA

 1140  TCCTTTTTGATAATCTCATGACCAAAATCCCTTAACGTGAGTTTTCGTTCCACTGAGCGT

 1200  CAGACCCCGTAGAAAAGATCAAAGGATCTTCTTGAGATCCTTTTTTTCTGCGCGTAATCT

 1260  GCTGCTTGCAAACAAAAAAACCACCGCTACCAGCGGTGGTTTGTTTGCCGGATCAAGAGC

 1320  TACCAACTCTTTTTCCGAAGGTAACTGGCTTCAGCAGAGCGCAGATACCAAATACTGTCC

 1380  TTCTAGTGTAGCCGTAGTTAGGCCACCACTTCAAGAACTCTGTAGCACCGCCTACATACC

 1440  TCGCTCTGCTAATCCTGTTACCAGTGGCTGCTGCCAGTGGCGATAAGTCGTGTCTTACCG

 1500  GGTTGGACTCAAGACGATAGTTACCGGATAAGGCGCAGCGGTCGGGCTGAACGGGGGGTT

 1560  CGTGCACACAGCCCAGCTTGGAGCGAACGACCTACACCGAACTGAGATACCTACAGCGTG

 1620  AGCTATGAGAAAGCGCCACGCTTCCCGAAGGGAGAAAGGCGGACAGGTATCCGGTAAGCG

 1680  GCAGGGTCGGAACAGGAGAGCGCACGAGGGAGCTTCCAGGGGGAAACGCCTGGTATCTTT

 1740  ATAGTCCTGTCGGGTTTCGCCACCTCTGACTTGAGCGTCGATTTTTGTGATGCTCGTCAG

 1800  GGGGGCGGAGCCTATGGAAAAACGCCAGCAACGCGGCCTTTTTACGGTTCCTGGCCTTTT

 1860  GCTGGCCTTTTGCTCACATGTTCTTTCCTGCGTTATCCCCTGATTCTGTGGATAACCGTA

 1920  TTACCGCCTTTGAGTGAGCTGATACCGCTCGCCGCAGCCGAACGACCGAGCGCAGCGAGT

 1980  CAGTGAGCGAGGAAGCGGAAGAGCGCCCAATACGCAAACCGCCTCTCCCCGCGCGTTGGC

 2040  CGATTCATTAATGCAGCTGGCACGACAGGTTTCCCGACTGGAAAGCGGGCAGTGAGCGCA

 2100  ACGCAATTAATGTGAGTTAGCTCACTCATTAGGCACCCCAGGCTTTACACTTTATGCTTC

 2160  CGGCTCGTATGTTGTGTGGAATTGTGAGCGGATAACAATTTCACACAGGAAACAGCTATG

*HindIII*

SceI **EcoRI** BamHI

 2220  ACCATGATTACGCCAAGCTCTAGGGATAACAGGGTAATCGAT**GAATTC***AAGCTT*GGAT**CC**

*q a w i*

**XmaI** KpnI

**SmaI**  **3 x FLAG**           *EcoRV*

 2280  **CGGG**TACC**GACTACAAAGACCATGACGGTGATTATAAAGATCAT*GATATC*GACTACAAAG**

*p g t* D Y K D H D G D Y K D H D I D Y K

**2 x Stop** Flp1

 2340  **ATGACGACGATAAATAGTAA**TGTAGGCTGGAGCTGCTTCGAAGTTCCTATACTTTCTAGA

  D D D D K * *

 2400  GAATAGGAACTTCGGAATAGGAACTTCAAGATCCCCCACGCTGCCGCAAGCACTCAGGGC

 2460  GCAAGGGCTGCTAAAGGAAGCGGAACACGTAGAAAGCCAGTCCGCAGAAACGGTGCTGAC

 2520  CCCGGATGAATGTCAGCTACTGGGCTATCTGGACAAGGGAAATCGCAAGCGCAAAGAGAA

 2580  AGCAGGTAGCTTGCAGTGGGCTTACATGGCGATAGCTAGACTGGGCGGTTTTATGGACAG

 2640  CAAGCGAACCGGAATTGCCAGCTGGGGCGCCCTCTGGTAAGGTTGGGAAGCCCTGCAAAG

 2700  TAAACTGGATGGCTTTCTTGCCGCCAAGGATCTGATGGCGCAGGGGATCAAGATCTGATC

 2760  AAGAGACAGGATGAGGATCGTTTCGCATGATTGAACAAGATGGATTGCACGCAGGTTCTC

    3                            **M**  I  E  Q  D  G  L  H  A  G  S  P

**Kan >**

 2820  CGGCCGCTTGGGTGGAGAGGCTATTCGGCTATGACTGGGCACAACAGACAATCGGCTGCT

    3    A  A  W  V  E  R  L  F  G  Y  D  W  A  Q  Q  T  I  G  C  S

 2880  CTGATGCCGCCGTGTTCCGGCTGTCAGCGCAGGGGCGCCCGGTTCTTTTTGTCAAGACCG

    3    D  A  A  V  F  R  L  S  A  Q  G  R  P  V  L  F  V  K  T  D

 2940  ACCTGTCCGGTGCCCTGAATGAACTGCAGGACGAGGCAGCGCGGCTATCGTGGCTGGCCA

    3    L  S  G  A  L  N  E  L  Q  D  E  A  A  R  L  S  W  L  A  T

 3000  CGACGGGCGTTCCTTGCGCAGCTGTGCTCGACGTTGTCACTGAAGCGGGAAGGGACTGGC

    3    T  G  V  P  C  A  A  V  L  D  V  V  T  E  A  G  R  D  W  L

 3060  TGCTATTGGGCGAAGTGCCGGGGCAGGATCTCCTGTCATCTCACCTTGCTCCTGCCGAGA

    3    L  L  G  E  V  P  G  Q  D  L  L  S  S  H  L  A  P  A  E  K

 3120  AAGTATCCATCATGGCTGATGCAATGCGGCGGCTGCATACGCTTGATCCGGCTACCTGCC

    3    V  S  I  M  A  D  A  M  R  R  L  H  T  L  D  P  A  T  C  P

 3180  CATTCGACCACCAAGCGAAACATCGCATCGAGCGAGCACGTACTCGGATGGAAGCCGGTC

    3    F  D  H  Q  A  K  H  R  I  E  R  A  R  T  R  M  E  A  G  L

 3240  TTGTCGATCAGGATGATCTGGACGAAGAGCATCAGGGGCTCGCGCCAGCCGAACTGTTCG

    3    V  D  Q  D  D  L  D  E  E  H  Q  G  L  A  P  A  E  L  F  A

 3300  CCAGGCTCAAGGCGCGCATGCCCGACGGCGAGGATCTCGTCGTGACCCATGGCGATGCCT

    3    R  L  K  A  R  M  P  D  G  E  D  L  V  V  T  H  G  D  A  C

 3360  GCTTGCCGAATATCATGGTGGAAAATGGCCGCTTTTCTGGATTCATCGACTGTGGCCGGC

    3    L  P  N  I  M  V  E  N  G  R  F  S  G  F  I  D  C  G  R  L

 3420  TGGGTGTGGCGGACCGCTATCAGGACATAGCGTTGGCTACCCGTGATATTGCTGAAGAGC

    3    G  V  A  D  R  Y  Q  D  I  A  L  A  T  R  D  I  A  E  E  L

 3480  TTGGCGGCGAATGGGCTGACCGCTTCCTCGTGCTCTACGGTATCGCCGCTCCCGATTCGC

    3    G  G  E  W  A  D  R  F  L  V  L  Y  G  I  A  A  P  D  S  Q

 3540  AGCGCATCGCCTTCTATCGCCTTCTTGACGAGTTCTTCTGAGCGGGACTCTGGGGTTCGA

    3    R  I  A  F  Y  R  L  L  D  E  F  F

 3600  AATGACCGACCAAGCGACGCCCAACCTGCCATCACGAGATTTCGATTCCACCGCCGCCTT

 3660  CTATGAAAGGTTGGGCTTCGGAATCGTTTTCCGGGACGCCGGCTGGATGATCCTCCAGCG

 3720  CGGGGATCTCATGCTGGAGTTCTTCGCCCACCCCAGCTTCAAAAGCGCTCTGAAGTTCCT

                                                                        Flp2 XhoI

 3780  ATACTTTCTAGAGAATAGGAACTTCGGAATAGGAACTAAGGAGGATATTCATATCTCGAG

**SacI**   **NheI**    *SpeI*

*NdeI*       SalI         SceI

 3840  **CT*C****ATAT****G*CTAGC**GTCG*ACTAGT*AGGGATAACAGGGTAATGAGCTTGGCACTGGCCGTCG

 3900  TTTTACAACGTCGTGACTGGGAAAACCCTGGCGTTACCCAACTTAATCGCCTTGCAGCAC

 3960  ATCCCCCTTTCGCCAGCTGGCGTAATAGCGAAGAGGCCCGCACCGATCGCCCTTCCCAAC

 4020  AGTTGCGCAGCCTGAATGGCGAATGGCGAGCTTGGCTGTTTTGGCGGATGAGAGAAGATT

 4080  TTCAGCCTGATACAGATTAAATCAGAACGCAGAAGCGGTCTGATAAAACAGAATTTGCCT

 4140  GGCGGCAGTAGCGCGGTGGTCCCACCTGACCCCATGCCGAACTCAGAAGTGAAACGCCGT

 4200  AGCGCCGATGGTAGTGTGGGGTCTCCCCATGCGAGAGTAGGGAACTGCCAGGCATCAAAT

 4260  AAAACGAAAGGCTCAGTCGAAAGACTGGGCCTTTCGTTTTATCTGTTGTTTGTCGGTGAA

 4320  CGCTCTCCTGAGTAGGACAAATCCGCCGGGAGCGGATTTGAACGTTGCGAAGCAACGGCC

 4380  CGGAGGGTGGCGGGCAGGACGCCCGCCATAAACTGCCAGGCATCAAATTAAGCAGAAGGC

 4440  CATCCTGACGGATGGCCTTTTTGCGTTTCTACAAACTCTTTTTGTTTATTTTTCTAAATA

 4500  CATTCAAATATGCATGCGCCTGATGCGGTATTTTCTCCTTACGCATATCGACATCCGCCC

 4560  TCACCGCCAGGAACGCAACCGCAGCCTCATCACGCCGGCGCTTCTTGGCCGCGCGGGATT

 4620  CAACCCACTCGGCCAGCTCGTCGGTGTAGCTCTTTGGCATCGTCTCTCGCCTGTCCCCTC

 4680  AGTTCAGTAATTTCCTGCATTTGCCTGTTTCCAGTCGGTAGATATTCCACAAAACAGCAG

 4740  GGAAGCAGCGCTTTTCCGCTGCATAACCCTGCTTCGGGGTCATTATAGCGATTTTTTCGG

 4800  TATATCCATCCTTTTTCGCACGATATACAGGATTTTGCCAAAGGGTTCGTGTAGACTTTC

 4860  CTTGGTGTATCCAACGGCGTCAGCCGGGCAGGATAGGTGAAGTAGGCCCACCCGCGAGCG

 4920  GGTGTTCCTTCTTCACTGTCCCTTATTCGCACCTGGCGGTGCTCAACGGGAATCCTGCTC

 4980  TGCGAGGCTGGCCGGCTACCGCCGGCGTAACAGATGAGGGCAAGCGGATGGCTGATGAAA

 5040  CCAAGCCAACCAGGAAGGGCAGCCCACCTATCAAGGTGTACTGCCTTCCAGACGAACGAA

 5100  GAGCGATTGAGGAAAAGGCGGCGGCGGCCGGCATGAGCCTGTCGGCCTACCTGCTGGCCG

 5160  TCGGCCAGGGCTACAAAATCACGGGCGTCGTGGACTATGAGCACGTCCGCGAGCTGGCCC

 5220  GCATCAATGGCGACCTGGGCCGCCTGGGCGGCCTGCTGAAACTCTGGCTCACCGACGACC

 5280  CGCGCACGGCGCGGTTCGGTGATGCCACGATCCTCGCCCTGCTGGCGAAGATCGACTCTA

 5340  GCTAGAGGATCGATCCTTTTTAACCCATCACATATACCTGCCGTTCACTATTATTTAGTG

 5400  AAATGAGATATTATGATATTTTCTGAATTGTGATTAAAAAGGCAACTTTATGCCCATGCA

 5460  ACAGAAACTATAAAAAATACAGAGAATGAAAAGAAACAGATAGATTTTTTAGTTCTTTAG

 5520  GCCCGTAGTCTGCAAATCCTTTTATGATTTTCTATCAAACAAAAGAGGAAAATAGACCAG

 5580  TTGCAATCCAAACGAGAGTCTAATAGAATGAGGTCGAAAAGTAAATCGCGCGGGTTTGTT

 5640  ACTGATAAAGCAGGCAAGACCTAAAATGTGTAAAGGGCAAAGTGTATACTTTGGCGTCAC

 5700  CCCTTACATATTTTAGGTCTTTTTTTATTGTGCGTAACTAACTTGCCATCTTCAAACAGG

 5760  AGGGCTGGAAGAAGCAGACCGCTAACACAGTACATAAAAAAGGAGACATGAACGATGAAC

    1                                                        M  N

**sacB >>**

 5820  ATCAAAAAGTTTGCAAAACAAGCAACAGTATTAACCTTTACTACCGCACTGCTGGCAGGA

    1  I  K  K  F  A  K  Q  A  T  V  L  T  F  T  T  A  L  L  A  G

 5880  GGCGCAACTCAAGCGTTTGCGAAAGAAACGAACCAAAAGCCATATAAGGAAACATACGGC

    1  G  A  T  Q  A  F  A  K  E  T  N  Q  K  P  Y  K  E  T  Y  G

 5940  ATTTCCCATATTACACGCCATGATATGCTGCAAATCCCTGAACAGCAAAAAAATGAAAAA

    1  I  S  H  I  T  R  H  D  M  L  Q  I  P  E  Q  Q  K  N  E  K

 6000  TATCAAGTTCCTGAGTTCGATTCGTCCACAATTAAAAATATCTCTTCTGCAAAAGGCCTG

    1  Y  Q  V  P  E  F  D  S  S  T  I  K  N  I  S  S  A  K  G  L

 6060  GACGTTTGGGACAGCTGGCCATTACAAAACGCTGACGGCACTGTCGCAAACTATCACGGC

    1  D  V  W  D  S  W  P  L  Q  N  A  D  G  T  V  A  N  Y  H  G

 6120  TACCACATCGTCTTTGCATTAGCCGGAGATCCTAAAAATGCGGATGACACATCGATTTAC

    1  Y  H  I  V  F  A  L  A  G  D  P  K  N  A  D  D  T  S  I  Y

 6180  ATGTTCTATCAAAAAGTCGGCGAAACTTCTATTGACAGCTGGAAAAACGCTGGCCGCGTC

    1  M  F  Y  Q  K  V  G  E  T  S  I  D  S  W  K  N  A  G  R  V

 6240  TTTAAAGACAGCGACAAATTCGATGCAAATGATTCTATCCTAAAAGACCAAACACAAGAA

    1  F  K  D  S  D  K  F  D  A  N  D  S  I  L  K  D  Q  T  Q  E

 6300  TGGTCAGGTTCAGCCACATTTACATCTGACGGAAAAATCCGTTTATTCTACACTGATTTC

    1  W  S  G  S  A  T  F  T  S  D  G  K  I  R  L  F  Y  T  D  F

 6360  TCCGGTAAACATTACGGCAAACAAACACTGACAACTGCACAAGTTAACGTATCAGCATCA

    1  S  G  K  H  Y  G  K  Q  T  L  T  T  A  Q  V  N  V  S  A  S

 6420  GACAGCTCTTTGAACATCAACGGTGTAGAGGATTATAAATCAATCTTTGACGGTGACGGA

    1  D  S  S  L  N  I  N  G  V  E  D  Y  K  S  I  F  D  G  D  G

 6480  AAAACGTATCAAAATGTACAGCAGTTCATCGATGAAGGCAACTACAGCTCAGGCGACAAC

    1  K  T  Y  Q  N  V  Q  Q  F  I  D  E  G  N  Y  S  S  G  D  N

 6540  CATACGCTGAGAGATCCTCACTACGTAGAAGATAAAGGCCACAAATACTTAGTATTTGAA

    1  H  T  L  R  D  P  H  Y  V  E  D  K  G  H  K  Y  L  V  F  E

 6600  GCAAACACTGGAACTGAAGATGGCTACCAAGGCGAAGAATCTTTATTTAACAAAGCATAC

    1  A  N  T  G  T  E  D  G  Y  Q  G  E  E  S  L  F  N  K  A  Y

 6660  TATGGCAAAAGCACATCATTCTTCCGTCAAGAAAGTCAAAAACTTCTGCAAAGCGATAAA

    1  Y  G  K  S  T  S  F  F  R  Q  E  S  Q  K  L  L  Q  S  D  K

 6720  AAACGCACGGCTGAGTTAGCAAACGGCGCTCTCGGTATGATTGAGCTAAACGATGATTAC

    1  K  R  T  A  E  L  A  N  G  A  L  G  M  I  E  L  N  D  D  Y

 6780  ACACTGAAAAAAGTGATGAAACCGCTGATTGCATCTAACACAGTAACAGATGAAATTGAA

    1  T  L  K  K  V  M  K  P  L  I  A  S  N  T  V  T  D  E  I  E

 6840  CGCGCGAACGTCTTTAAAATGAACGGCAAATGGTATCTGTTCACTGACTCCCGCGGATCA

    1  R  A  N  V  F  K  M  N  G  K  W  Y  L  F  T  D  S  R  G  S

 6900  AAAATGACGATTGACGGCATTACGTCTAACGATATTTACATGCTTGGTTATGTTTCTAAT

    1  K  M  T  I  D  G  I  T  S  N  D  I  Y  M  L  G  Y  V  S  N

 6960  TCTTTAACTGGCCCATACAAGCCGCTGAACAAAACTGGCCTTGTGTTAAAAATGGATCTT

    1  S  L  T  G  P  Y  K  P  L  N  K  T  G  L  V  L  K  M  D  L

 7020  GATCCTAACGATGTAACCTTTACTTACTCACACTTCGCTGTACCTCAAGCGAAAGGAAAC

    1  D  P  N  D  V  T  F  T  Y  S  H  F  A  V  P  Q  A  K  G  N

 7080  AATGTCGTGATTACAAGCTATATGACAAACAGAGGATTCTACGCAGACAAACAATCAACG

    1  N  V  V  I  T  S  Y  M  T  N  R  G  F  Y  A  D  K  Q  S  T

 7140  TTTGCGCCTAGCTTCCTGCTGAACATCAAAGGCAAGAAAACATCTGTTGTCAAAGACAGC

    1  F  A  P  S  F  L  L  N  I  K  G  K  K  T  S  V  V  K  D  S

 7200  ATCCTTGAACAAGGACAATTAACAGTTAACAAATAAAAACGCAAAAGAAAATGCCGATTA

    1  I  L  E  Q  G  Q  L  T  V  N  K

 7260  TGGTGCACTCTCAGTACAATCTGCTCTGATGCCGCATAGTTAAGCCAGCCCCGACACCCG

 7320  CCAACACCCGCTGACGCGCCCTGACGGGCTTGTCTGCTCCCGGCATCCGCTTACAGACAA

 7380  GCTGTGACCGTCTCCGGGAGCTGCATGTGTCAGAGGTTTTCACCGTCATCACCGAAACGC

 7440  GCGA

**pDOC-P**

0  GACGAAAGGGCCTCGTGATACGCCTATTTTTATAGGTTAATGTCATGATAATAATGGTTT

   60  CTTAGACGTCAGGTGGCACTTTTCGGGGAAATGTGCGCGGAACCCCTATTTGTTTATTTT

  120  TCTAAATACATTCAAATATGTATCCGCTCATGAGACAATAACCCTGATAAATGCTTCAAT

  180  AATATTGAAAAAGGAAGAGTATGAGTATTCAACATTTCCGTGTCGCCCTTATTCCCTTTT

    3                      M  S  I  Q  H  F  R  V  A  L  I  P  F  F

**bla >>**

  240  TTGCGGCATTTTGCCTTCCTGTTTTTGCTCACCCAGAAACGCTGGTGAAAGTAAAAGATG

    3    A  A  F  C  L  P  V  F  A  H  P  E  T  L  V  K  V  K  D  A

  300  CTGAAGATCAGTTGGGTGCACGAGTGGGTTACATCGAACTGGATCTCAACAGCGGTAAGA

    3    E  D  Q  L  G  A  R  V  G  Y  I  E  L  D  L  N  S  G  K  I

  360  TCCTTGAGAGTTTTCGCCCCGAAGAACGTTTTCCAATGATGAGCACTTTTAAAGTTCTGC

    3    L  E  S  F  R  P  E  E  R  F  P  M  M  S  T  F  K  V  L  L

  420  TATGTGGCGCGGTATTATCCCGTATTGACGCCGGGCAAGAGCAACTCGGTCGCCGCATAC

    3    C  G  A  V  L  S  R  I  D  A  G  Q  E  Q  L  G  R  R  I  H

  480  ACTATTCTCAGAATGACTTGGTTGAGTACTCACCAGTCACAGAAAAGCATCTTACGGATG

    3    Y  S  Q  N  D  L  V  E  Y  S  P  V  T  E  K  H  L  T  D  G

  540  GCATGACAGTAAGAGAATTATGCAGTGCTGCCATAACCATGAGTGATAACACTGCGGCCA

    3    M  T  V  R  E  L  C  S  A  A  I  T  M  S  D  N  T  A  A  N

  600  ACTTACTTCTGACAACGATCGGAGGACCGAAGGAGCTAACCGCTTTTTTGCACAACATGG

    3    L  L  L  T  T  I  G  G  P  K  E  L  T  A  F  L  H  N  M  G

  660  GGGATCATGTAACTCGCCTTGATCGTTGGGAACCGGAGCTGAATGAAGCCATACCAAACG

    3    D  H  V  T  R  L  D  R  W  E  P  E  L  N  E  A  I  P  N  D

  720  ACGAGCGTGACACCACGATGCCTGTAGCAATGGCAACAACGTTGCGCAAACTATTAACTG

    3    E  R  D  T  T  M  P  V  A  M  A  T  T  L  R  K  L  L  T  G

  780  GCGAACTACTTACTCTAGCTTCCCGGCAACAATTAATAGACTGGATGGAGGCGGATAAAG

    3    E  L  L  T  L  A  S  R  Q  Q  L  I  D  W  M  E  A  D  K  V

  840  TTGCAGGACCACTTCTGCGCTCGGCCCTTCCGGCTGGCTGGTTTATTGCTGATAAATCTG

    3    A  G  P  L  L  R  S  A  L  P  A  G  W  F  I  A  D  K  S  G

  900  GAGCCGGTGAGCGTGGGTCTCGCGGTATCATTGCAGCACTGGGGCCAGATGGTAAGCCCT

    3    A  G  E  R  G  S  R  G  I  I  A  A  L  G  P  D  G  K  P  S

  960  CCCGTATCGTAGTTATCTACACGACGGGGAGTCAGGCAACTATGGATGAACGAAATAGAC

    3    R  I  V  V  I  Y  T  T  G  S  Q  A  T  M  D  E  R  N  R  Q

 1020  AGATCGCTGAGATAGGTGCCTCACTGATTAAGCATTGGTAACTGTCAGACCAAGTTTACT

    3    I  A  E  I  G  A  S  L  I  K  H  W

 1080  CATATATACTTTAGATTGATTTAAAACTTCATTTTTAATTTAAAAGGATCTAGGTGAAGA

 1140  TCCTTTTTGATAATCTCATGACCAAAATCCCTTAACGTGAGTTTTCGTTCCACTGAGCGT

 1200  CAGACCCCGTAGAAAAGATCAAAGGATCTTCTTGAGATCCTTTTTTTCTGCGCGTAATCT

 1260  GCTGCTTGCAAACAAAAAAACCACCGCTACCAGCGGTGGTTTGTTTGCCGGATCAAGAGC

 1320  TACCAACTCTTTTTCCGAAGGTAACTGGCTTCAGCAGAGCGCAGATACCAAATACTGTCC

 1380  TTCTAGTGTAGCCGTAGTTAGGCCACCACTTCAAGAACTCTGTAGCACCGCCTACATACC

 1440  TCGCTCTGCTAATCCTGTTACCAGTGGCTGCTGCCAGTGGCGATAAGTCGTGTCTTACCG

 1500  GGTTGGACTCAAGACGATAGTTACCGGATAAGGCGCAGCGGTCGGGCTGAACGGGGGGTT

 1560  CGTGCACACAGCCCAGCTTGGAGCGAACGACCTACACCGAACTGAGATACCTACAGCGTG

 1620  AGCTATGAGAAAGCGCCACGCTTCCCGAAGGGAGAAAGGCGGACAGGTATCCGGTAAGCG

 1680  GCAGGGTCGGAACAGGAGAGCGCACGAGGGAGCTTCCAGGGGGAAACGCCTGGTATCTTT

 1740  ATAGTCCTGTCGGGTTTCGCCACCTCTGACTTGAGCGTCGATTTTTGTGATGCTCGTCAG

 1800  GGGGGCGGAGCCTATGGAAAAACGCCAGCAACGCGGCCTTTTTACGGTTCCTGGCCTTTT

 1860  GCTGGCCTTTTGCTCACATGTTCTTTCCTGCGTTATCCCCTGATTCTGTGGATAACCGTA

 1920  TTACCGCCTTTGAGTGAGCTGATACCGCTCGCCGCAGCCGAACGACCGAGCGCAGCGAGT

 1980  CAGTGAGCGAGGAAGCGGAAGAGCGCCCAATACGCAAACCGCCTCTCCCCGCGCGTTGGC

 2040  CGATTCATTAATGCAGCTGGCACGACAGGTTTCCCGACTGGAAAGCGGGCAGTGAGCGCA

 2100  ACGCAATTAATGTGAGTTAGCTCACTCATTAGGCACCCCAGGCTTTACACTTTATGCTTC

 2160  CGGCTCGTATGTTGTGTGGAATTGTGAGCGGATAACAATTTCACACAGGAAACAGCTATG

*HindIII*

                                SceI             **EcoRI**       BamHI

 2220  ACCATGATTACGCCAAGCTCTAGGGATAACAGGGTAATCGAT**GAATTC***AAGCTTG*GAT**CC**

**XmaI**

**SmaI**  KpnI   **Protein A Start**

 2280  **CGGG**TACC**AGC**GGTGAAGCTCAAAAACTTAATGACTCTCAAGCTCCAAAAGCTGATGCGC

  S G E A Q K L N D S Q A P K A D A Q

 2340  AACAAAATAACTTCAACAAAGATCAACAAAGCGCCTTCTATGAAATCTTGAACATGCCTA

  Q N N F N K D Q Q S A F Y E I L N M P N

 2400  ACTTAAACGAAGCGCAACGTAACGGCTTCATTCAAAGTCTTAAAGACGACCCAAGCCAAA

  L N E A Q R N G F I Q S L K D D P S Q S

 2460  GCACTAACGTTTTAGGTGAAGCTAAAAAATTAAACGAATCTCAAGCACCGAAAGCTGATA

  T N V L G E A K K L N E S Q A P K A D N

 2520  ACAATTTCAACAAAGAACAACAAAATGCTTTCTATGAAATCTTGAATATGCCTAACTTAA

N F N K E Q Q N A F Y E I L N M P N L N

                                    HindIII

 2580  ACGAAGAACAACGCAATGGTTTCATCCAAAGCTTAAAAGATGACCCGAGCCAAAGTGCTA

  E E Q R N G F I Q S L K D D P S Q S A N

 2640  ACCTATTGTCAGAAGCTAAAAAGTTAAATGAATCTCAAGCACCGAAAGCGGATAACAAAT

  L L S E A K K L N E S Q A P K A D N K F

 2700  TCAACAAAGAACAACAAAATGCTTTCTATGAAATCTTACATTTACCTAACTTAAACGAAG

  N K E Q Q N A F Y E I L H L P N L N E E

 2760  AACAACGCAATGGTTTCATCCAAAGCCTAAAAGATGACCCAAGCCAAAGCGCTAACCTTT

  Q R N G F I Q S L K D D P S Q S A N L L

 2820  TAGCAGAAGCTAAAAAGCTAAATGATGCTCAAGCACCAAAAGCTGACAACAAATTCAACA

  A E A K K L N D A Q A P K A D N K F N K

 2880  AAGAACAACAAAATGCTTTCTATGAAATTTTACATTTACCTAACTTAACTGAAGAACAAC

  E Q Q N A F Y E I L H L P N L T E E Q R

                                           STOP   **AgeI**  MunI

 2940  GTAACGGCTTCATCCAAAGCCTTAAAGACGATGCCGGG**TAA**TGT**ACCGGT**CAATTGGCTG

N G F I Q S L K D D A G

flp

 3000  GAGCTGCTTCGAAGTTCCTATACTTTCTAGAGAATAGGAACTTCGGAATAGGAACTTCAA

 3060  GATCCCCCACGCTGCCGCAAGCACTCAGGGCGCAAGGGCTGCTAAAGGAAGCGGAACACG

 3120  TAGAACTTAAGGGAATTGCCAGCTGGGGCGCCCTCTGGTAAGGTTGGGAAGCCCTGCAAA

 3180  GTAAACTGGATGGCTTTCTTGCCGCCAAGGATCTGATGGCGCAGGGGATCAAGATCTGAT

 3240  CAAGAGACAGGATGAGGATCGTTTCGCATGATTGAACAAGATGGATTGCACGCAGGTTCT

    1                             **M**  I  E  Q  D  G  L  H  A  G  S

**Kan >**

 3300  CCGGCCGCTTGGGTGGAGAGGCTATTCGGCTATGACTGGGCACAACAGACAATCGGCTGC

    1  P  A  A  W  V  E  R  L  F  G  Y  D  W  A  Q  Q  T  I  G  C

 3360  TCTGATGCCGCCGTGTTCCGGCTGTCAGCGCAGGGGCGCCCGGTTCTTTTTGTCAAGACC

    1  S  D  A  A  V  F  R  L  S  A  Q  G  R  P  V  L  F  V  K  T

 3420  GACCTGTCCGGTGCCCTGAATGAACTGCAGGACGAGGCAGCGCGGCTATCGTGGCTGGCC

    1  D  L  S  G  A  L  N  E  L  Q  D  E  A  A  R  L  S  W  L  A

 3480  ACGACGGGCGTTCCTTGCGCAGCTGTGCTCGACGTTGTCACTGAAGCGGGAAGGGACTGG

    1  T  T  G  V  P  C  A  A  V  L  D  V  V  T  E  A  G  R  D  W

 3540  CTGCTATTGGGCGAAGTGCCGGGGCAGGATCTCCTGTCATCTCACCTTGCTCCTGCCGAG

    1  L  L  L  G  E  V  P  G  Q  D  L  L  S  S  H  L  A  P  A  E

 3600  AAAGTATCCATCATGGCTGATGCAATGCGGCGGCTGCATACGCTTGATCCGGCTACCTGC

    1  K  V  S  I  M  A  D  A  M  R  R  L  H  T  L  D  P  A  T  C

 3660  CCATTCGACCACCAAGCGAAACATCGCATCGAGCGAGCACGTACTCGGATGGAAGCCGGT

    1  P  F  D  H  Q  A  K  H  R  I  E  R  A  R  T  R  M  E  A  G

 3720  CTTGTCGATCAGGATGATCTGGACGAAGAGCATCAGGGGCTCGCGCCAGCCGAACTGTTC

    1  L  V  D  Q  D  D  L  D  E  E  H  Q  G  L  A  P  A  E  L  F

 3780  GCCAGGCTCAAGGCGCGCATGCCCGACGGCGAGGATCTCGTCGTGACCCATGGCGATGCC

    1  A  R  L  K  A  R  M  P  D  G  E  D  L  V  V  T  H  G  D  A

 3840  TGCTTGCCGAATATCATGGTGGAAAATGGCCGCTTTTCTGGATTCATCGACTGTGGCCGG

    1  C  L  P  N  I  M  V  E  N  G  R  F  S  G  F  I  D  C  G  R

 3900  CTGGGTGTGGCGGACCGCTATCAGGACATAGCGTTGGCTACCCGTGATATTGCTGAAGAG

    1  L  G  V  A  D  R  Y  Q  D  I  A  L  A  T  R  D  I  A  E  E

 3960  CTTGGCGGCGAATGGGCTGACCGCTTCCTCGTGCTCTACGGTATCGCCGCTCCCGATTCG

    1  L  G  G  E  W  A  D  R  F  L  V  L  Y  G  I  A  A  P  D  S

 4020  CAGCGCATCGCCTTCTATCGCCTTCTTGACGAGTTCTTCTGAGCGGGACTCTGGGGTTCG

    1  Q  R  I  A  F  Y  R  L  L  D  E  F  F

 4080  AAATGACCGACCAAGCGACGCCCAACCTGCCATCACGAGATTTCGATTCCACCGCCGCCT

 4140  TCTATGAAAGGTTGGGCTTCGGAATCGTTTTCCGGGACGCCGGCTGGATGATCCTCCAGC

 4200  GCGGGGATCTCATGCTGGAGTTCTTCGCCCACCCCAGCTTCAAAAGCGCTCTGAAGTTCC

                                                                        Flp2 XhoI

 4260  TATACTTTCTAGAGAATAGGAACTTCGGAATAGGAACTAAGGAGGATATTCATATCTC**GA**

**SacI**   **NheI**    *SpeI*             **XmaI**

*NdeI*       SalI         SceI    **SmaI**

 4320  **GCT*C****ATAT****G*CTAGC**GTCG*ACTAGT*AGGGATAACAGGGTAAT**CCCGGG**TAGGGATAACAGG

 4380  GTAATGAGCTTGGCACTGGCCGTCGTTTTACAACGTCGTGACTGGGAAAACCCTGGCGTT

 4440  ACCCAACTTAATCGCCTTGCAGCACATCCCCCTTTCGCCAGCTGGCGTAATAGCGAAGAG

 4500  GCCCGCACCGATCGCCCTTCCCAACAGTTGCGCAGCCTGAATGGCGAATGGCGAGCTTGG

 4560  CTGTTTTGGCGGATGAGAGAAGATTTTCAGCCTGATACAGATTAAATCAGAACGCAGAAG

 4620  CGGTCTGATAAAACAGAATTTGCCTGGCGGCAGTAGCGCGGTGGTCCCACCTGACCCCAT

 4680  GCCGAACTCAGAAGTGAAACGCCGTAGCGCCGATGGTAGTGTGGGGTCTCCCCATGCGAG

 4740  AGTAGGGAACTGCCAGGCATCAAATAAAACGAAAGGCTCAGTCGAAAGACTGGGCCTTTC

 4800  GTTTTATCTGTTGTTTGTCGGTGAACGCTCTCCTGAGTAGGACAAATCCGCCGGGAGCGG

 4860  ATTTGAACGTTGCGAAGCAACGGCCCGGAGGGTGGCGGGCAGGACGCCCGCCATAAACTG

 4920  CCAGGCATCAAATTAAGCAGAAGGCCATCCTGACGGATGGCCTTTTTGCGTTTCTACAAA

 4980  CTCTTTTTGTTTATTTTTCTAAATACATTCAAATATGCATGCGCCTGATGCGGTATTTTC

 5040  TCCTTACGCATATCGACATCCGCCCTCACCGCCAGGAACGCAACCGCAGCCTCATCACGC

 5100  CGGCGCTTCTTGGCCGCGCGGGATTCAACCCACTCGGCCAGCTCGTCGGTGTAGCTCTTT

 5160  GGCATCGTCTCTCGCCTGTCCCCTCAGTTCAGTAATTTCCTGCATTTGCCTGTTTCCAGT

 5220  CGGTAGATATTCCACAAAACAGCAGGGAAGCAGCGCTTTTCCGCTGCATAACCCTGCTTC

 5280  GGGGTCATTATAGCGATTTTTTCGGTATATCCATCCTTTTTCGCACGATATACAGGATTT

 5340  TGCCAAAGGGTTCGTGTAGACTTTCCTTGGTGTATCCAACGGCGTCAGCCGGGCAGGATA

 5400  GGTGAAGTAGGCCCACCCGCGAGCGGGTGTTCCTTCTTCACTGTCCCTTATTCGCACCTG

 5460  GCGGTGCTCAACGGGAATCCTGCTCTGCGAGGCTGGCCGGCTACCGCCGGCGTAACAGAT

 5520  GAGGGCAAGCGGATGGCTGATGAAACCAAGCCAACCAGGAAGGGCAGCCCACCTATCAAG

 5580  GTGTACTGCCTTCCAGACGAACGAAGAGCGATTGAGGAAAAGGCGGCGGCGGCCGGCATG

 5640  AGCCTGTCGGCCTACCTGCTGGCCGTCGGCCAGGGCTACAAAATCACGGGCGTCGTGGAC

 5700  TATGAGCACGTCCGCGAGCTGGCCCGCATCAATGGCGACCTGGGCCGCCTGGGCGGCCTG

 5760  CTGAAACTCTGGCTCACCGACGACCCGCGCACGGCGCGGTTCGGTGATGCCACGATCCTC

 5820  GCCCTGCTGGCGAAGATCGACTCTAGCTAGAGGATCGATCCTTTTTAACCCATCACATAT

 5880  ACCTGCCGTTCACTATTATTTAGTGAAATGAGATATTATGATATTTTCTGAATTGTGATT

 5940  AAAAAGGCAACTTTATGCCCATGCAACAGAAACTATAAAAAATACAGAGAATGAAAAGAA

 6000  ACAGATAGATTTTTTAGTTCTTTAGGCCCGTAGTCTGCAAATCCTTTTATGATTTTCTAT

 6060  CAAACAAAAGAGGAAAATAGACCAGTTGCAATCCAAACGAGAGTCTAATAGAATGAGGTC

 6120  GAAAAGTAAATCGCGCGGGTTTGTTACTGATAAAGCAGGCAAGACCTAAAATGTGTAAAG

 6180  GGCAAAGTGTATACTTTGGCGTCACCCCTTACATATTTTAGGTCTTTTTTTATTGTGCGT

 6240  AACTAACTTGCCATCTTCAAACAGGAGGGCTGGAAGAAGCAGACCGCTAACACAGTACAT

 6300  AAAAAAGGAGACATGAACGATGAACATCAAAAAGTTTGCAAAACAAGCAACAGTATTAAC

    2                     M  N  I  K  K  F  A  K  Q  A  T  V  L  T

**sacB >>**

 6360  CTTTACTACCGCACTGCTGGCAGGAGGCGCAACTCAAGCGTTTGCGAAAGAAACGAACCA

    2   F  T  T  A  L  L  A  G  G  A  T  Q  A  F  A  K  E  T  N  Q

 6420  AAAGCCATATAAGGAAACATACGGCATTTCCCATATTACACGCCATGATATGCTGCAAAT

    2   K  P  Y  K  E  T  Y  G  I  S  H  I  T  R  H  D  M  L  Q  I

 6480  CCCTGAACAGCAAAAAAATGAAAAATATCAAGTTCCTGAGTTCGATTCGTCCACAATTAA

    2   P  E  Q  Q  K  N  E  K  Y  Q  V  P  E  F  D  S  S  T  I  K

 6540  AAATATCTCTTCTGCAAAAGGCCTGGACGTTTGGGACAGCTGGCCATTACAAAACGCTGA

    2   N  I  S  S  A  K  G  L  D  V  W  D  S  W  P  L  Q  N  A  D

 6600  CGGCACTGTCGCAAACTATCACGGCTACCACATCGTCTTTGCATTAGCCGGAGATCCTAA

    2   G  T  V  A  N  Y  H  G  Y  H  I  V  F  A  L  A  G  D  P  K

 6660  AAATGCGGATGACACATCGATTTACATGTTCTATCAAAAAGTCGGCGAAACTTCTATTGA

    2   N  A  D  D  T  S  I  Y  M  F  Y  Q  K  V  G  E  T  S  I  D

 6720  CAGCTGGAAAAACGCTGGCCGCGTCTTTAAAGACAGCGACAAATTCGATGCAAATGATTC

    2   S  W  K  N  A  G  R  V  F  K  D  S  D  K  F  D  A  N  D  S

 6780  TATCCTAAAAGACCAAACACAAGAATGGTCAGGTTCAGCCACATTTACATCTGACGGAAA

    2   I  L  K  D  Q  T  Q  E  W  S  G  S  A  T  F  T  S  D  G  K

 6840  AATCCGTTTATTCTACACTGATTTCTCCGGTAAACATTACGGCAAACAAACACTGACAAC

    2   I  R  L  F  Y  T  D  F  S  G  K  H  Y  G  K  Q  T  L  T  T

 6900  TGCACAAGTTAACGTATCAGCATCAGACAGCTCTTTGAACATCAACGGTGTAGAGGATTA

    2   A  Q  V  N  V  S  A  S  D  S  S  L  N  I  N  G  V  E  D  Y

 6960  TAAATCAATCTTTGACGGTGACGGAAAAACGTATCAAAATGTACAGCAGTTCATCGATGA

    2   K  S  I  F  D  G  D  G  K  T  Y  Q  N  V  Q  Q  F  I  D  E

 7020  AGGCAACTACAGCTCAGGCGACAACCATACGCTGAGAGATCCTCACTACGTAGAAGATAA

    2   G  N  Y  S  S  G  D  N  H  T  L  R  D  P  H  Y  V  E  D  K

 7080  AGGCCACAAATACTTAGTATTTGAAGCAAACACTGGAACTGAAGATGGCTACCAAGGCGA

    2   G  H  K  Y  L  V  F  E  A  N  T  G  T  E  D  G  Y  Q  G  E

 7140  AGAATCTTTATTTAACAAAGCATACTATGGCAAAAGCACATCATTCTTCCGTCAAGAAAG

    2   E  S  L  F  N  K  A  Y  Y  G  K  S  T  S  F  F  R  Q  E  S

 7200  TCAAAAACTTCTGCAAAGCGATAAAAAACGCACGGCTGAGTTAGCAAACGGCGCTCTCGG

    2   Q  K  L  L  Q  S  D  K  K  R  T  A  E  L  A  N  G  A  L  G

 7260  TATGATTGAGCTAAACGATGATTACACACTGAAAAAAGTGATGAAACCGCTGATTGCATC

    2   M  I  E  L  N  D  D  Y  T  L  K  K  V  M  K  P  L  I  A  S

 7320  TAACACAGTAACAGATGAAATTGAACGCGCGAACGTCTTTAAAATGAACGGCAAATGGTA

    2   N  T  V  T  D  E  I  E  R  A  N  V  F  K  M  N  G  K  W  Y

 7380  TCTGTTCACTGACTCCCGCGGATCAAAAATGACGATTGACGGCATTACGTCTAACGATAT

    2   L  F  T  D  S  R  G  S  K  M  T  I  D  G  I  T  S  N  D  I

 7440  TTACATGCTTGGTTATGTTTCTAATTCTTTAACTGGCCCATACAAGCCGCTGAACAAAAC

    2   Y  M  L  G  Y  V  S  N  S  L  T  G  P  Y  K  P  L  N  K  T

 7500  TGGCCTTGTGTTAAAAATGGATCTTGATCCTAACGATGTAACCTTTACTTACTCACACTT

    2   G  L  V  L  K  M  D  L  D  P  N  D  V  T  F  T  Y  S  H  F

 7560  CGCTGTACCTCAAGCGAAAGGAAACAATGTCGTGATTACAAGCTATATGACAAACAGAGG

    2   A  V  P  Q  A  K  G  N  N  V  V  I  T  S  Y  M  T  N  R  G

 7620  ATTCTACGCAGACAAACAATCAACGTTTGCGCCTAGCTTCCTGCTGAACATCAAAGGCAA

    2   F  Y  A  D  K  Q  S  T  F  A  P  S  F  L  L  N  I  K  G  K

 7680  GAAAACATCTGTTGTCAAAGACAGCATCCTTGAACAAGGACAATTAACAGTTAACAAATA

    2   K  T  S  V  V  K  D  S  I  L  E  Q  G  Q  L  T  V  N  K

 7740  AAAACGCAAAAGAAAATGCCGATTATGGTGCACTCTCAGTACAATCTGCTCTGATGCCGC

 7800  ATAGTTAAGCCAGCCCCGACACCCGCCAACACCCGCTGACGCGCCCTGACGGGCTTGTCT

 7860  GCTCCCGGCATCCGCTTACAGACAAGCTGTGACCGTCTCCGGGAGCTGCATGTGTCAGAG

 7920  GTTTTCACCGTCATCACCGAAACGCGCGA

**pDOC-G**

0  GACGAAAGGGCCTCGTGATACGCCTATTTTTATAGGTTAATGTCATGATAATAATGGTTT

   60  CTTAGACGTCAGGTGGCACTTTTCGGGGAAATGTGCGCGGAACCCCTATTTGTTTATTTT

  120  TCTAAATACATTCAAATATGTATCCGCTCATGAGACAATAACCCTGATAAATGCTTCAAT

  180  AATATTGAAAAAGGAAGAGTATGAGTATTCAACATTTCCGTGTCGCCCTTATTCCCTTTT

    3                      M  S  I  Q  H  F  R  V  A  L  I  P  F  F

**bla >>**

  240  TTGCGGCATTTTGCCTTCCTGTTTTTGCTCACCCAGAAACGCTGGTGAAAGTAAAAGATG

    3    A  A  F  C  L  P  V  F  A  H  P  E  T  L  V  K  V  K  D  A

  300  CTGAAGATCAGTTGGGTGCACGAGTGGGTTACATCGAACTGGATCTCAACAGCGGTAAGA

    3    E  D  Q  L  G  A  R  V  G  Y  I  E  L  D  L  N  S  G  K  I

  360  TCCTTGAGAGTTTTCGCCCCGAAGAACGTTTTCCAATGATGAGCACTTTTAAAGTTCTGC

    3    L  E  S  F  R  P  E  E  R  F  P  M  M  S  T  F  K  V  L  L

  420  TATGTGGCGCGGTATTATCCCGTATTGACGCCGGGCAAGAGCAACTCGGTCGCCGCATAC

    3    C  G  A  V  L  S  R  I  D  A  G  Q  E  Q  L  G  R  R  I  H

  480  ACTATTCTCAGAATGACTTGGTTGAGTACTCACCAGTCACAGAAAAGCATCTTACGGATG

    3    Y  S  Q  N  D  L  V  E  Y  S  P  V  T  E  K  H  L  T  D  G

  540  GCATGACAGTAAGAGAATTATGCAGTGCTGCCATAACCATGAGTGATAACACTGCGGCCA

    3    M  T  V  R  E  L  C  S  A  A  I  T  M  S  D  N  T  A  A  N

  600  ACTTACTTCTGACAACGATCGGAGGACCGAAGGAGCTAACCGCTTTTTTGCACAACATGG

    3    L  L  L  T  T  I  G  G  P  K  E  L  T  A  F  L  H  N  M  G

  660  GGGATCATGTAACTCGCCTTGATCGTTGGGAACCGGAGCTGAATGAAGCCATACCAAACG

    3    D  H  V  T  R  L  D  R  W  E  P  E  L  N  E  A  I  P  N  D

  720  ACGAGCGTGACACCACGATGCCTGTAGCAATGGCAACAACGTTGCGCAAACTATTAACTG

    3    E  R  D  T  T  M  P  V  A  M  A  T  T  L  R  K  L  L  T  G

  780  GCGAACTACTTACTCTAGCTTCCCGGCAACAATTAATAGACTGGATGGAGGCGGATAAAG

    3    E  L  L  T  L  A  S  R  Q  Q  L  I  D  W  M  E  A  D  K  V

  840  TTGCAGGACCACTTCTGCGCTCGGCCCTTCCGGCTGGCTGGTTTATTGCTGATAAATCTG

    3    A  G  P  L  L  R  S  A  L  P  A  G  W  F  I  A  D  K  S  G

  900  GAGCCGGTGAGCGTGGGTCTCGCGGTATCATTGCAGCACTGGGGCCAGATGGTAAGCCCT

    3    A  G  E  R  G  S  R  G  I  I  A  A  L  G  P  D  G  K  P  S

  960  CCCGTATCGTAGTTATCTACACGACGGGGAGTCAGGCAACTATGGATGAACGAAATAGAC

    3    R  I  V  V  I  Y  T  T  G  S  Q  A  T  M  D  E  R  N  R  Q

 1020  AGATCGCTGAGATAGGTGCCTCACTGATTAAGCATTGGTAACTGTCAGACCAAGTTTACT

    3    I  A  E  I  G  A  S  L  I  K  H  W

 1080  CATATATACTTTAGATTGATTTAAAACTTCATTTTTAATTTAAAAGGATCTAGGTGAAGA

 1140  TCCTTTTTGATAATCTCATGACCAAAATCCCTTAACGTGAGTTTTCGTTCCACTGAGCGT

 1200  CAGACCCCGTAGAAAAGATCAAAGGATCTTCTTGAGATCCTTTTTTTCTGCGCGTAATCT

 1260  GCTGCTTGCAAACAAAAAAACCACCGCTACCAGCGGTGGTTTGTTTGCCGGATCAAGAGC

 1320  TACCAACTCTTTTTCCGAAGGTAACTGGCTTCAGCAGAGCGCAGATACCAAATACTGTCC

 1380  TTCTAGTGTAGCCGTAGTTAGGCCACCACTTCAAGAACTCTGTAGCACCGCCTACATACC

 1440  TCGCTCTGCTAATCCTGTTACCAGTGGCTGCTGCCAGTGGCGATAAGTCGTGTCTTACCG

 1500  GGTTGGACTCAAGACGATAGTTACCGGATAAGGCGCAGCGGTCGGGCTGAACGGGGGGTT

 1560  CGTGCACACAGCCCAGCTTGGAGCGAACGACCTACACCGAACTGAGATACCTACAGCGTG

 1620  AGCTATGAGAAAGCGCCACGCTTCCCGAAGGGAGAAAGGCGGACAGGTATCCGGTAAGCG

 1680  GCAGGGTCGGAACAGGAGAGCGCACGAGGGAGCTTCCAGGGGGAAACGCCTGGTATCTTT

 1740  ATAGTCCTGTCGGGTTTCGCCACCTCTGACTTGAGCGTCGATTTTTGTGATGCTCGTCAG

 1800  GGGGGCGGAGCCTATGGAAAAACGCCAGCAACGCGGCCTTTTTACGGTTCCTGGCCTTTT

 1860  GCTGGCCTTTTGCTCACATGTTCTTTCCTGCGTTATCCCCTGATTCTGTGGATAACCGTA

 1920  TTACCGCCTTTGAGTGAGCTGATACCGCTCGCCGCAGCCGAACGACCGAGCGCAGCGAGT

 1980  CAGTGAGCGAGGAAGCGGAAGAGCGCCCAATACGCAAACCGCCTCTCCCCGCGCGTTGGC

 2040  CGATTCATTAATGCAGCTGGCACGACAGGTTTCCCGACTGGAAAGCGGGCAGTGAGCGCA

 2100  ACGCAATTAATGTGAGTTAGCTCACTCATTAGGCACCCCAGGCTTTACACTTTATGCTTC

 2160  CGGCTCGTATGTTGTGTGGAATTGTGAGCGGATAACAATTTCACACAGGAAACAGCTATG

*HindIII*

                                    SceI          **EcoRI**       BamHI

 2220  ACCATGATTACGCCAAGCTCTAGGGATAACAGGGTAATCGAT**GAATTC***AAGCTT*GGAT**CC**

**XmaI**

**SmaI** KpnI     **Gfp Start**

 2280  **CGGG**TACC**AGC**AAGGGCGAGGAGCTGTTCACCGGGGTGGTGCCCATCCTGGTCGAGCTGG

  S K G E E L F T G V V P I L V E L D

 2340  ACGGCGACGTAAACGGCCACAAGTTCAGCGTGTCCGGCGAGGGCGAGGGCGATGCCACCT

  G D V N G H K F S V S G E G E G D A T Y

 2400  ACGGCAAGCTGACCCTGAAGTTCATCTGCACCACCGGCAAGCTGCCCGTGCCCTGGCCCA

  G K L T L K F I C T T G K L P V P W P T

 2460  CCCTCGTGACCACCTTGACCTACGGCGTGCAGTGCTTCGCCCGCTACCCCGACCACATGA

  L V T T L T Y G V Q C F A R Y P D H M K

 2520  AGCAGCACGACTTCTTCAAGTCCGCCATGCCCGAAGGCTACGTCCAGGAGCGCACCATCT

  Q H D F F K S A M P E G Y V Q E R T I F

 2580  TCTTCAAGGACGACGGCAACTACAAGACCCGCGCCGAGGTGAAGTTCGAGGGCGACACCC

  F K D D G N Y K T R A E V K F E G D T L

 2640  TGGTGAACCGCATCGAGCTGAAGGGCATCGACTTCAAGGAGGACGGCAACATCCTGGGGC

  V N R I E L K G I D F K E D G N I L G H

 2700  ACAAGCTGGAGTACAACTACAACAGCCACAAGGTCTATATCACCGCCGACAAGCAGAAGA

  K L E Y N Y N S H K V Y I T A D K Q K N

 2760  ACGGCATCAAGGTGAACTTCAAGACCCGCCACAACATCGAGGACGGCAGCGTGCAGCTCG

  G I K V N F K T R H N I E D G S V Q L A

 2820  CCGACCACTACCAGCAGAACACCCCCATCGGCGACGGCCCCGTGCTGCTGCCCGACAACC

  D H Y Q Q N T P I G D G P V L L P D N H

 2880  ACTACCTGAGCACCCAGTCCGCCCTGAGCAAAGACCCCAACGAGAAGCGCGATCACATGG

  Y L S T Q S A L S K D P N E K R D H M V

 2940  TCCTGCTGGAGTTCGTGACCGCCGCCGGGATCACTCTCGGCATGGACGAGCTGTACAAGT

  L L E F V T A A G I T L G **M** D E L Y K

**AgeI**  MunI                      Flp1

 3000  AA**ACCGGT**CAATTGGCTGGAGCTGCTTCGAAGTTCCTATACTTTCTAGAGAATAGGAACT

**STOP**

 3060  TCGGAATAGGAACTTCAAGATCCCCCACGCTGCCGCAAGCACTCAGGGCGCAAGGGCTGC

 3120  TAAAGGAAGCGGAACACGTAGAACTTAAGGGAATTGCCAGCTGGGGCGCCCTCTGGTAAG

 3180  GTTGGGAAGCCCTGCAAAGTAAACTGGATGGCTTTCTTGCCGCCAAGGATCTGATGGCGC

 3240  AGGGGATCAAGATCTGATCAAGAGACAGGATGAGGATCGTTTCGCATGATTGAACAAGAT

    1                                               **M**  I  E  Q  D

**Kan >**

 3300  GGATTGCACGCAGGTTCTCCGGCCGCTTGGGTGGAGAGGCTATTCGGCTATGACTGGGCA

    1  G  L  H  A  G  S  P  A  A  W  V  E  R  L  F  G  Y  D  W  A

 3360  CAACAGACAATCGGCTGCTCTGATGCCGCCGTGTTCCGGCTGTCAGCGCAGGGGCGCCCG

    1  Q  Q  T  I  G  C  S  D  A  A  V  F  R  L  S  A  Q  G  R  P

 3420  GTTCTTTTTGTCAAGACCGACCTGTCCGGTGCCCTGAATGAACTGCAGGACGAGGCAGCG

    1  V  L  F  V  K  T  D  L  S  G  A  L  N  E  L  Q  D  E  A  A

 3480  CGGCTATCGTGGCTGGCCACGACGGGCGTTCCTTGCGCAGCTGTGCTCGACGTTGTCACT

    1  R  L  S  W  L  A  T  T  G  V  P  C  A  A  V  L  D  V  V  T

 3540  GAAGCGGGAAGGGACTGGCTGCTATTGGGCGAAGTGCCGGGGCAGGATCTCCTGTCATCT

    1  E  A  G  R  D  W  L  L  L  G  E  V  P  G  Q  D  L  L  S  S

 3600  CACCTTGCTCCTGCCGAGAAAGTATCCATCATGGCTGATGCAATGCGGCGGCTGCATACG

    1  H  L  A  P  A  E  K  V  S  I  M  A  D  A  M  R  R  L  H  T

 3660  CTTGATCCGGCTACCTGCCCATTCGACCACCAAGCGAAACATCGCATCGAGCGAGCACGT

    1  L  D  P  A  T  C  P  F  D  H  Q  A  K  H  R  I  E  R  A  R

 3720  ACTCGGATGGAAGCCGGTCTTGTCGATCAGGATGATCTGGACGAAGAGCATCAGGGGCTC

    1  T  R  M  E  A  G  L  V  D  Q  D  D  L  D  E  E  H  Q  G  L

 3780  GCGCCAGCCGAACTGTTCGCCAGGCTCAAGGCGCGCATGCCCGACGGCGAGGATCTCGTC

    1  A  P  A  E  L  F  A  R  L  K  A  R  M  P  D  G  E  D  L  V

 3840  GTGACCCATGGCGATGCCTGCTTGCCGAATATCATGGTGGAAAATGGCCGCTTTTCTGGA

    1  V  T  H  G  D  A  C  L  P  N  I  M  V  E  N  G  R  F  S  G

 3900  TTCATCGACTGTGGCCGGCTGGGTGTGGCGGACCGCTATCAGGACATAGCGTTGGCTACC

    1  F  I  D  C  G  R  L  G  V  A  D  R  Y  Q  D  I  A  L  A  T

 3960  CGTGATATTGCTGAAGAGCTTGGCGGCGAATGGGCTGACCGCTTCCTCGTGCTTTACGGT

    1  R  D  I  A  E  E  L  G  G  E  W  A  D  R  F  L  V  L  Y  G

 4020  ATCGCCGCTCCCGATTCGCAGCGCATCGCCTTCTATCGCCTTCTTGACGAGTTCTTCTGA

    1  I  A  A  P  D  S  Q  R  I  A  F  Y  R  L  L  D  E  F  F

 4080  GCGGGACTCTGGGGTTCGAAATGACCGACCAAGCGACGCCCAACCTGCCATCACGAGATT

 4140  TCGATTCCACCGCCGCCTTCTATGAAAGGTTGGGCTTCGGAATCGTTTTCCGGGACGCCG

 4200  GCTGGATGATCCTCCAGCGCGGGGATCTCATGCTGGAGTTCTTCGCCCACCCCAGCTTCA

  Flp2

 4260  AAAGCGCTCTGAAGTTCCTATACTTTCTAGAGAATAGGAACTTCGGAATAGGAACTAAGG

**SacI**   **NheI**    *SpeI*

                     XhoI    *NdeI*       SalI         SceI

 4320  AGGATATTCATATCTC**GAGCT*C****ATAT****G*CTAGC**GTCG*ACTAGT*AGGGATAACAGGGTAATG

 4380  AGCTTGGCACTGGCCGTCGTTTTACAACGTCGTGACTGGGAAAACCCTGGCGTTACCCAA

 4440  CTTAATCGCCTTGCAGCACATCCCCCTTTCGCCAGCTGGCGTAATAGCGAAGAGGCCCGC

 4500  ACCGATCGCCCTTCCCAACAGTTGCGCAGCCTGAATGGCGAATGGCGAGCTTGGCTGTTT

 4560  TGGCGGATGAGAGAAGATTTTCAGCCTGATACAGATTAAATCAGAACGCAGAAGCGGTCT

 4620  GATAAAACAGAATTTGCCTGGCGGCAGTAGCGCGGTGGTCCCACCTGACCCCATGCCGAA

 4680  CTCAGAAGTGAAACGCCGTAGCGCCGATGGTAGTGTGGGGTCTCCCCATGCGAGAGTAGG

 4740  GAACTGCCAGGCATCAAATAAAACGAAAGGCTCAGTCGAAAGACTGGGCCTTTCGTTTTA

 4800  TCTGTTGTTTGTCGGTGAACGCTCTCCTGAGTAGGACAAATCCGCCGGGAGCGGATTTGA

 4860  ACGTTGCGAAGCAACGGCCCGGAGGGTGGCGGGCAGGACGCCCGCCATAAACTGCCAGGC

 4920  ATCAAATTAAGCAGAAGGCCATCCTGACGGATGGCCTTTTTGCGTTTCTACAAACTCTTT

 4980  TTGTTTATTTTTCTAAATACATTCAAATATGCATGCGCCTGATGCGGTATTTTCTCCTTA

 5040  CGCATATCGACATCCGCCCTCACCGCCAGGAACGCAACCGCAGCCTCATCACGCCGGCGC

 5100  TTCTTGGCCGCGCGGGATTCAACCCACTCGGCCAGCTCGTCGGTGTAGCTCTTTGGCATC

 5160  GTCTCTCGCCTGTCCCCTCAGTTCAGTAATTTCCTGCATTTGCCTGTTTCCAGTCGGTAG

 5220  ATATTCCACAAAACAGCAGGGAAGCAGCGCTTTTCCGCTGCATAACCCTGCTTCGGGGTC

 5280  ATTATAGCGATTTTTTCGGTATATCCATCCTTTTTCGCACGATATACAGGATTTTGCCAA

 5340  AGGGTTCGTGTAGACTTTCCTTGGTGTATCCAACGGCGTCAGCCGGGCAGGATAGGTGAA

 5400  GTAGGCCCACCCGCGAGCGGGTGTTCCTTCTTCACTGTCCCTTATTCGCACCTGGCGGTG

 5460  CTCAACGGGAATCCTGCTCTGCGAGGCTGGCCGGCTACCGCCGGCGTAACAGATGAGGGC

 5520  AAGCGGATGGCTGATGAAACCAAGCCAACCAGGAAGGGCAGCCCACCTATCAAGGTGTAC

 5580  TGCCTTCCAGACGAACGAAGAGCGATTGAGGAAAAGGCGGCGGCGGCCGGCATGAGCCTG

 5640  TCGGCCTACCTGCTGGCCGTCGGCCAGGGCTACAAAATCACGGGCGTCGTGGACTATGAG

 5700  CACGTCCGCGAGCTGGCCCGCATCAATGGCGACCTGGGCCGCCTGGGCGGCCTGCTGAAA

 5760  CTCTGGCTCACCGACGACCCGCGCACGGCGCGGTTCGGTGATGCCACGATCCTCGCCCTG

 5820  CTGGCGAAGATCGACTCTAGCTAGAGGATCGATCCTTTTTAACCCATCACATATACCTGC

 5880  CGTTCACTATTATTTAGTGAAATGAGATATTATGATATTTTCTGAATTGTGATTAAAAAG

 5940  GCAACTTTATGCCCATGCAACAGAAACTATAAAAAATACAGAGAATGAAAAGAAACAGAT

 6000  AGATTTTTTAGTTCTTTAGGCCCGTAGTCTGCAAATCCTTTTATGATTTTCTATCAAACA

 6060  AAAGAGGAAAATAGACCAGTTGCAATCCAAACGAGAGTCTAATAGAATGAGGTCGAAAAG

 6120  TAAATCGCGCGGGTTTGTTACTGATAAAGCAGGCAAGACCTAAAATGTGTAAAGGGCAAA

 6180  GTGTATACTTTGGCGTCACCCCTTACATATTTTAGGTCTTTTTTTATTGTGCGTAACTAA

 6240  CTTGCCATCTTCAAACAGGAGGGCTGGAAGAAGCAGACCGCTAACACAGTACATAAAAAA

 6300  GGAGACATGAACGATGAACATCAAAAAGTTTGCAAAACAAGCAACAGTATTAACCTTTAC

    2               M  N  I  K  K  F  A  K  Q  A  T  V  L  T  F  T

**sacB >>**

 6360  TACCGCACTGCTGGCAGGAGGCGCAACTCAAGCGTTTGCGAAAGAAACGAACCAAAAGCC

    2   T  A  L  L  A  G  G  A  T  Q  A  F  A  K  E  T  N  Q  K  P

 6420  ATATAAGGAAACATACGGCATTTCCCATATTACACGCCATGATATGCTGCAAATCCCTGA

    2   Y  K  E  T  Y  G  I  S  H  I  T  R  H  D  M  L  Q  I  P  E

 6480  ACAGCAAAAAAATGAAAAATATCAAGTTCCTGAGTTCGATTCGTCCACAATTAAAAATAT

    2   Q  Q  K  N  E  K  Y  Q  V  P  E  F  D  S  S  T  I  K  N  I

 6540  CTCTTCTGCAAAAGGCCTGGACGTTTGGGACAGCTGGCCATTACAAAACGCTGACGGCAC

    2   S  S  A  K  G  L  D  V  W  D  S  W  P  L  Q  N  A  D  G  T

 6600  TGTCGCAAACTATCACGGCTACCACATCGTCTTTGCATTAGCCGGAGATCCTAAAAATGC

    2   V  A  N  Y  H  G  Y  H  I  V  F  A  L  A  G  D  P  K  N  A

 6660  GGATGACACATCGATTTACATGTTCTATCAAAAAGTCGGCGAAACTTCTATTGACAGCTG

    2   D  D  T  S  I  Y  M  F  Y  Q  K  V  G  E  T  S  I  D  S  W

 6720  GAAAAACGCTGGCCGCGTCTTTAAAGACAGCGACAAATTCGATGCAAATGATTCTATCCT

    2   K  N  A  G  R  V  F  K  D  S  D  K  F  D  A  N  D  S  I  L

 6780  AAAAGACCAAACACAAGAATGGTCAGGTTCAGCCACATTTACATCTGACGGAAAAATCCG

    2   K  D  Q  T  Q  E  W  S  G  S  A  T  F  T  S  D  G  K  I  R

 6840  TTTATTCTACACTGATTTCTCCGGTAAACATTACGGCAAACAAACACTGACAACTGCACA

    2   L  F  Y  T  D  F  S  G  K  H  Y  G  K  Q  T  L  T  T  A  Q

 6900  AGTTAACGTATCAGCATCAGACAGCTCTTTGAACATCAACGGTGTAGAGGATTATAAATC

    2   V  N  V  S  A  S  D  S  S  L  N  I  N  G  V  E  D  Y  K  S

 6960  AATCTTTGACGGTGACGGAAAAACGTATCAAAATGTACAGCAGTTCATCGATGAAGGCAA

    2   I  F  D  G  D  G  K  T  Y  Q  N  V  Q  Q  F  I  D  E  G  N

 7020  CTACAGCTCAGGCGACAACCATACGCTGAGAGATCCTCACTACGTAGAAGATAAAGGCCA

    2   Y  S  S  G  D  N  H  T  L  R  D  P  H  Y  V  E  D  K  G  H

 7080  CAAATACTTAGTATTTGAAGCAAACACTGGAACTGAAGATGGCTACCAAGGCGAAGAATC

    2   K  Y  L  V  F  E  A  N  T  G  T  E  D  G  Y  Q  G  E  E  S

 7140  TTTATTTAACAAAGCATACTATGGCAAAAGCACATCATTCTTCCGTCAAGAAAGTCAAAA

    2   L  F  N  K  A  Y  Y  G  K  S  T  S  F  F  R  Q  E  S  Q  K

 7200  ACTTCTGCAAAGCGATAAAAAACGCACGGCTGAGTTAGCAAACGGCGCTCTCGGTATGAT

    2   L  L  Q  S  D  K  K  R  T  A  E  L  A  N  G  A  L  G  M  I

 7260  TGAGCTAAACGATGATTACACACTGAAAAAAGTGATGAAACCGCTGATTGCATCTAACAC

    2   E  L  N  D  D  Y  T  L  K  K  V  M  K  P  L  I  A  S  N  T

 7320  AGTAACAGATGAAATTGAACGCGCGAACGTCTTTAAAATGAACGGCAAATGGTATCTGTT

    2   V  T  D  E  I  E  R  A  N  V  F  K  M  N  G  K  W  Y  L  F

 7380  CACTGACTCCCGCGGATCAAAAATGACGATTGACGGCATTACGTCTAACGATATTTACAT

    2   T  D  S  R  G  S  K  M  T  I  D  G  I  T  S  N  D  I  Y  M

 7440  GCTTGGTTATGTTTCTAATTCTTTAACTGGCCCATACAAGCCGCTGAACAAAACTGGCCT

    2   L  G  Y  V  S  N  S  L  T  G  P  Y  K  P  L  N  K  T  G  L

 7500  TGTGTTAAAAATGGATCTTGATCCTAACGATGTAACCTTTACTTACTCACACTTCGCTGT

    2   V  L  K  M  D  L  D  P  N  D  V  T  F  T  Y  S  H  F  A  V

 7560  ACCTCAAGCGAAAGGAAACAATGTCGTGATTACAAGCTATATGACAAACAGAGGATTCTA

    2   P  Q  A  K  G  N  N  V  V  I  T  S  Y  M  T  N  R  G  F  Y

 7620  CGCAGACAAACAATCAACGTTTGCGCCTAGCTTCCTGCTGAACATCAAAGGCAAGAAAAC

    2   A  D  K  Q  S  T  F  A  P  S  F  L  L  N  I  K  G  K  K  T

 7680  ATCTGTTGTCAAAGACAGCATCCTTGAACAAGGACAATTAACAGTTAACAAATAAAAACG

    2   S  V  V  K  D  S  I  L  E  Q  G  Q  L  T  V  N  K

 7740  CAAAAGAAAATGCCGATTATGGTGCACTCTCAGTACAATCTGCTCTGATGCCGCATAGTT

 7800  AAGCCAGCCCCGACACCCGCCAACACCCGCTGACGCGCCCTGACGGGCTTGTCTGCTCCC

 7860  GGCATCCGCTTACAGACAAGCTGTGACCGTCTCCGGGAGCTGCATGTGTCAGAGGTTTTC

 7920  ACCGTCATCACCGAAACGCGCGA
